# The multifaceted role of SsrB in *S*. Typhi H58 during THP-1 macrophage infection

**DOI:** 10.3389/fmicb.2026.1849419

**Published:** 2026-10-12

**Authors:** Marion Fernandez, Soham Deolankar, Moirangthem Kiran Singh, Parisa Zangoui, Linda J. Kenney

**Affiliations:** 1 Department of Biochemistry and Molecular Biology, University of Texas Medical Branch, Galveston, TX, United States; 2 Department of Chemistry, Dhanamanjuri University, Imphal, Manipur, India; 3 Mechanobiologics, Inc., Santa Fe, NM, United States

**Keywords:** acid pH, *Salmonella* Typhi, SPI-2, SSRB, two-component signaling, typhoid fever

## Abstract

*S*. Typhi, the causative agent of typhoid fever, remains less well understood due to its human-restricted nature compared to *S*. Typhimurium, which enjoys a broad host range. Herein, we investigated the acid response of *S*. Typhi and the role of the SPI-2 master regulator SsrB during infection of human THP-1 macrophages. Using fluorescence lifetime imaging of the pH-sensitive probe mCherryTYG, we demonstrate that the *S*. Typhi cytosol acidifies during infection of THP-1 macrophages, similar to *S*. Typhimurium. This acidification triggers a marked up-regulation of SsrB, with *S*. Typhi expressing significantly more SsrB molecules than *S*. Typhimurium, as quantified by photoactivatable localization microscopy. Despite increased SsrB expression, activation of the SsrB-dependent reporter P*sifA* was reduced, indicating serovar-specific differences in SsrB activity. Unexpectedly, deletion of *ssrB* in *S*. Typhi increased net intracellular replication and host cell cytotoxicity, contrasting with its essential role in *S*. Typhimurium. Using phosphorylation incompetent (D56A) and acid-sensing deficient (H12Q) mutants, we demonstrate that the reduction in cytotoxicity is phosphorylation-independent, whereas restriction of net intracellular replication requires both phosphorylation and acid-dependent DNA binding. The SPI-2 effector SifB partially mediates this restriction. Our findings show that SsrB contributes to two infection phenotypes with distinct genetic requirements: it limits host cytotoxicity in a manner that requires neither phosphorylation nor acid-enhanced DNA binding, and it restricts intracellular replication in a manner that requires both activities. Whether these two phenotypes are executed by separate or by shared downstream targets was not established here. These results challenge the assumption that SsrB is dispensable for *S*. Typhi pathogenesis and highlight serovar-specific virulence strategies.

## 1 Introduction

*Salmonella enterica* serovar Typhi is the causative agent of typhoid fever, a human-restricted, severe systemic infection. Typhoid fever is mainly present in Asia as well as in Africa, where it results in approximately 10 million cases per year, with an associated 110,000 deaths (Hancuh et al., 2023; Meiring and Johnston, 2023). In contrast, *Salmonella enterica* serovar Typhimurium (STm) causes gastroenteritis, and can infect a large range of hosts such as humans, poultry or cattle (Eng et al., 2015). Because of its human restricted nature, studies of *S*.Typhi (STy) are challenging and most research has focused on *S*. Typhimurium.

Following ingestion of contaminated food or water, *Salmonella* survives the acidic pH of the stomach and reaches the intestine. *Salmonella* is capable of invading and crossing the intestinal epithelial barrier by the expression of *Salmonella* pathogenicity island 1 (SPI-1), encoding a type three secretion system (T3SS) and its associated effectors (Cirillo et al., 1998; Galán and Curtiss, 1989). *Salmonella* is subsequently phagocytosed by immune cells such as macrophages. During its intracellular life, *Salmonella* resides in a *Salmonella-*containing vacuole (SCV) (Steele-Mortimer, 2008), a hostile environment, where it survives by activation of SPI-2, encoding a second (T3SS) and secretion of its associated effectors (Hensel et al., 1998). The acidic environment of the vacuole is a major signal that triggers activation of SPI-2 in *S*Tm (Kenney, 2019). In the acidic environment of the SCV, *S*Tm acidifies its own cytosol, catalyzed by the OmpR/EnvZ two-component signaling system (Chakraborty et al., 2017, 2015). Bacterial acidification later triggers the activation of SsrB (Liew et al., 2019), the two-component system regulating SPI-2, through a conserved pH-sensitive histidine residue in the phosphorylation domain. Acid pH-activation of SsrB increases the DNA binding affinity of SsrB for SPI-2 genes by more than 30-fold (Shetty and Kenney, 2023).

*S*. Typhi and *S*. Typhimurium share approximately 80% of their genes, which has made it tempting to extrapolate the Typhimurium model to Typhi. The two serovars nonetheless differ markedly in their pathogenesis, and the molecular basis of this specificity is poorly understood. The master regulator of SPI-2, SsrB, is essential for *S*. Typhimurium to survive and replicate within host cells, whereas studies of its role in *S*. Typhi have produced apparently divergent findings and left many remaining questions. Previous studies reported that SsrB-dependent SPI-2 expression was very limited in *S*. Typhi, in comparison to *S*. Typhimurium (Forest et al., 2010). In parallel, gentamicin protection approaches with an *ssrB* null strain failed to resolve a phenotype using a THP-1 macrophage model (Forest et al., 2010; Murret-Labarthe et al., 2020). In contrast, a study following *S*. Typhi replication in THP-1 cells using fluorescence dilution methods reported a growth defect for the SsrB target gene *sifA*. That study did not investigate, but raised questions concerning the role of this master regulator (Hamblin et al., 2023).

Herein, we investigated the intracellular acid response of *S*. Typhi and the role of SsrB in the human macrophage THP-1 model. We first tracked the intracellular pH (pHi) of *S*. Typhi in response to an acidic environment, using our recently published Fluorescence Lifetime Imaging (FLIM)-based pH sensor (Singh et al., 2025). We highlight that, similar to *S*. Typhimurium, the *S*. Typhi cytosol acidifies in the acidic compartment of the SCV. Through extensive microscopic analysis of strains lacking *ssrB* or expressing phosphorylation-incompetent (SsrB^D56A^) or pH-sensing (SsrB^H12Q^) mutants, we demonstrate that SsrB is required for two infection phenotypes with distinct genetic requirements. Unphosphorylated SsrB is required to mitigate bacterial cytotoxicity toward the host cell, whereas the acid-induced response requiring SsrB phosphorylation and high-affinity DNA binding is essential for restricting net intracellular replication. Interestingly, despite robust activation of SsrB production within macrophages, we demonstrate that activation of the SsrB-dependent *sifA* promoter is reduced in *S*. Typhi compared to *S*. Typhimurium, despite a higher abundance of SsrB. This reduction in SsrB activation is a direct result of its more oxidizing intracellular environment (Fernandez et al., 2025). In summary, our work establishes SsrB as a critical factor that finely modulates the interaction of *S*. Typhi with human macrophages by controlling both net intracellular replication and host cell cytotoxicity.

## 2 Materials and methods

### 2.1 Bacterial strains and plasmids

For the deletion of *ssrB* and *prgHIJ* in *S*. Typhi, genes of interest were replaced by the chloramphenicol cassette (CmR), amplified from pKD3 with primers listed in Supplementary Table S1 by λ-Red recombination with pKD46 (Datsenko and Wanner, 2000). Mutants were selected on LB agar supplemented with 30 μg/mL chloramphenicol. Deletions were verified using internal and deletion flanking primers. *ssrB*^D56A^ and *ssrB*^H12Q^ strain were constructed by integration of the corresponding allele fused to a chloramphenicol cassette at the *ssrB* locus of the *ssrB* null strain, previously cured by pCP20. Both alleles are single-codon chromosomal substitutions integrated at the native *ssrB* locus, expressed from the native promoter and 5' untranslated region, with an identical downstream cassette architecture in the two strains. Neither was plasmid-borne or overexpressed, so the two mutants differ from each other only by the substituted codon. The H58 SsrB-PAmCherry strain was made by transduction from the 14028s SsrB-PAmCherry strain (Liew et al., 2019). For constitutive expression of *sifB*, the gene was amplified by PCR from the H58 genome and cloned onto the low copy plasmid pSC101 with an expression driven by the *ybaJp* promoter. pFPVmCherryTYG and p*cadBA*, pZS::P*sifA-*mTagBFP, P*Te*t-mCherry and 14028s *ssrB*::*kan* are lab collections.

### 2.2 Infection of THP-1 macrophages

THP-1 monocytes (ATCC TIB-202) were maintained in RPMI-1640 medium supplemented with 10% fetal bovine serum (Opti-Gold, Gene Depot), 2 mM L-glutamine (Sigma-Aldrich, Merck KGaA, Darmstadt, Germany), 1% MEM amino acid solution (Gibco, Thermo Fisher Scientific, Waltham, MA, USA), 1 mM sodium pyruvate (Sigma), and 1% penicillin-streptomycin (Gene Depot, GenDEPOT, Barker, TX, USA). Cultures were incubated at 37 °C in a humidified atmosphere containing 5% CO_2_. For imaging experiments, phenol red-free RPMI was used. To induce macrophage differentiation, cells were seeded at a density of 1 × 10^5^ per well in either 24-well plates (Corning, Corning Inc., Corning, NY, USA) for CFU assays or 8-well chamber slides (Sarstedt, Germany) for microscopy, and treated with 10 μM phorbol 12-myristate 13-acetate (PMA; Sigma) for 48 h. *Salmonella* Typhi was cultured by inoculating a single colony into 3 mL LB broth and incubating statically overnight (~16 h) at 37 °C until reaching an OD_600_ of approximately 0.6. Differentiated macrophages were infected at a multiplicity of infection (MOI) of 10 (or MOI 5 + 5 for co-infections). To synchronize bacterial uptake, plates were centrifuged at 600 × g for 5 min, followed by a 25 min infection period. Cells were then washed with DPBS containing 100 μg/mL gentamicin and incubated for 30 min in RPMI supplemented with 100 μg/mL gentamicin to kill extracellular bacteria. Subsequently, the medium was replaced with RPMI containing 12 μg/mL gentamicin for the remainder of the experiment (defined as time zero, T0). Gentamicin was used solely for standard post-infection extracellular killing (100 μg/mL for 30 min) and subsequently maintained at 12 μg/mL to prevent outgrowth of extracellular bacteria. Macrophages were not subjected to long-term antibiotic pre-treatment. The centrifugation at 600 × g for 5 min was solely to synchronize initial contact, and subsequent washing plus a defined MOI of 10 ensured comparable bacterial inputs across conditions. We did not observe evidence of overt cell damage or altered baseline responses attributable to centrifugation alone. To assess bacterial uptake, some wells were lysed immediately after the 30 min gentamicin treatment. Throughout, uptake refers to bacterial counts at 0.5 hpi, that is, immediately after the 30 min gentamicin treatment; intracellular burden refers to the number of bacteria per infected cell at a given time point; and net intracellular replication refers to the ratio of counts at 24 hpi to counts at 0.5 hpi. The last is a net measure and does not distinguish an increased rate of division from reduced bacterial killing. It should be noted that bacterial counts per cell were determined exclusively for intracellular bacteria after extracellular bacteria had been eliminated by gentamicin treatment. The infected cells were washed twice with PBS and lysed with 0.1% Triton X-100 for 10 min at 30 min post-infection. The lysates were serially diluted and plated on LB agar and incubated overnight for counting, to obtain the intracellular colony-forming units (CFUs) (Hoffmann et al., 2018; García-Armesto et al., 1993). For microscopy imaging, cells were seeded into glass-bottom eight-well chambers (Sarstedt) and maintained in phenol red-free RPMI medium. Where indicated, infected cells were fixed at the specified time points using 4% paraformaldehyde (Thermo Fisher, Thermo Fisher Scientific, Waltham, MA, USA) for 10 min at room temperature. “Bacteria per infected THP-1 cell” refers to intracellular bacteria only, quantified by confocal microscopy on fixed, permeabilized cells after extracellular killing with gentamicin and extensive washing. For Figure 1 and other single-cell analyses, bacteria were counted in confocal images and each dot in the figures corresponds to one infected THP-1 cell.

**Figure 1:**
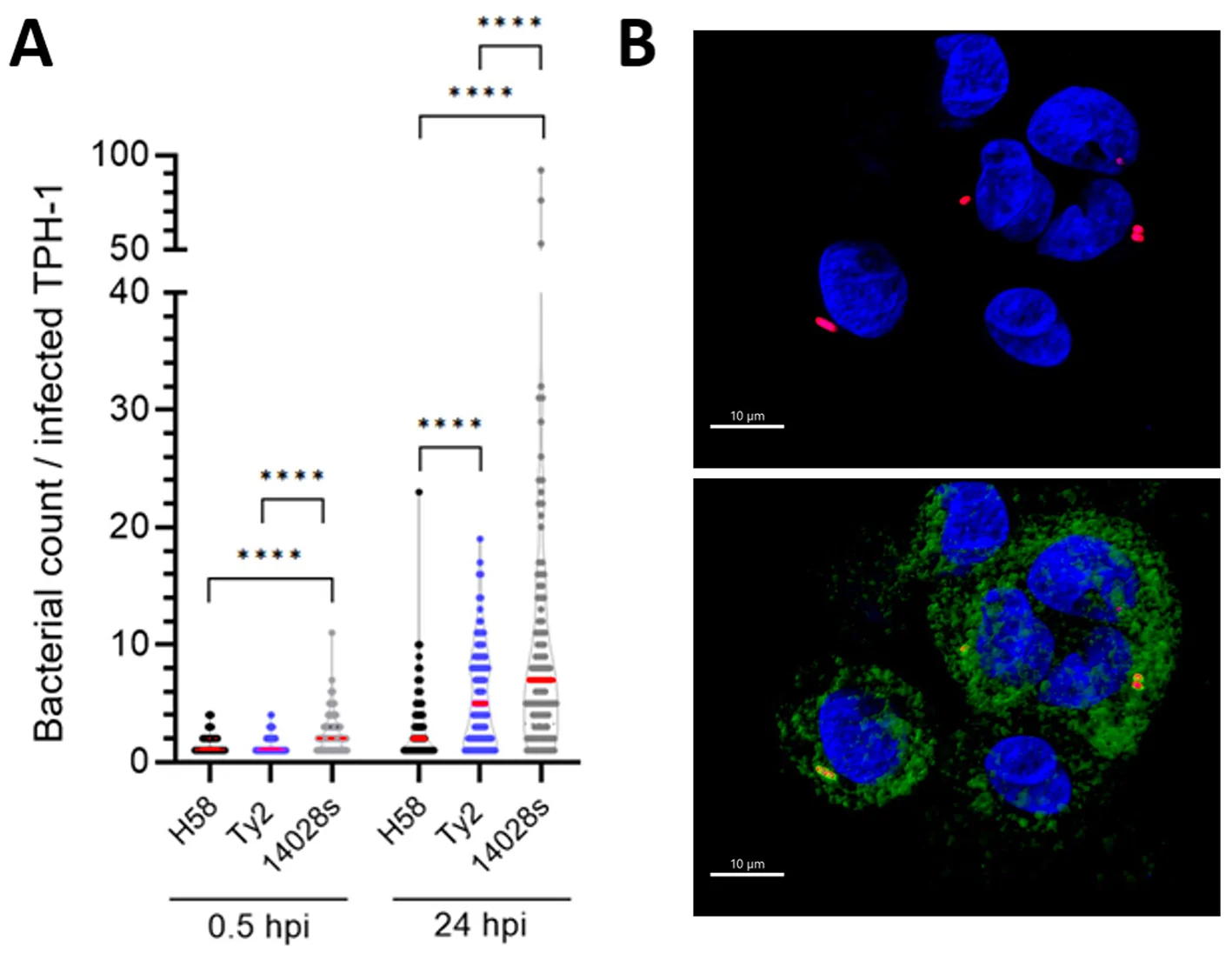
*S*. Typhi displays a low net intracellular replication in THP-1 macrophages. THP-1 macrophages were infected with *Salmonella* strains expressing pFPV-mCherry. **(A)** *S*Tm reaches a much higher intracellular burden than *S*Ty strains in THP-1 macrophages. The number of bacteria per infected THP-1 was enumerated by microscopy at 0.5 and 24 hpi. Each dot represents one infected THP-1 cell with *n* = 100 cells per condition, from three independent experiments. Statistical significance was determined using One-Way ANOVA (****p < 0.0001). **(B)** THP-1 cells infected with *S*Ty H58 were fixed with 4% PFA at 24 hpi and LAMP-1 was labeled by immunostaining. The immunostaining was qualitative and performed at 24 hpi only. Blue, DAPI; Red, *S*. Typhi pFPV-mCherry; Green, LAMP-1. Images shown in this figure are representative selections from datasets used for the corresponding quantification. Scale bar: 10 μm.

### 2.3 LDH assays

Cytotoxicity of bacteria toward the macrophage host was assessed using the CytoTox 96^®^ Non-Radioactive Cytotoxicity Assay (Promega, Promega Corporation, Madison, WI, USA) following manufacturer's instructions. Briefly, measures were performed in the supernatant of infected or uninfected THP-1 macrophages at the indicated time point. The sample absorbance at 490 nm was determined and the value was converted to a cytotoxicity percentage using a maximum lysis control.

### 2.4 *Salmonella* pH_*i*_ measurements using BCECF

To generate the *in vitro* standard curve, the free acid form of BCECF (Invitrogen) was incubated in buffers ranging from pH 5.0 to 7.2 in 96 well plate (Costar). Bacteria were immobilized on agarose pads, ensuring that cell motility did not interfere with image acquisition. Fluorescence intensities were measured using excitation wavelengths of 488 and 440 nm, with emission recorded at 525 nm using a Tecan spectrophotometer (GENios). To generate the *in vivo* standard curve, exponential phase bacteria were stained with 20 μM of BCECF and clamped in 100 mM potassium phosphate buffer at various pH containing 40 μM nigericin. Bacteria were seeded onto agarose pads, and Imaging was performed on a Leica TCS SP8 STED 3X confocal system (Wetzlar, Germany) outfitted with an HC PL APO CS2 100 × /1.40 oil immersion objective. Paired images were acquired using excitation at 440 nm, corresponding to the pH-insensitive wavelength, and 488 nm, the pH-sensitive wavelength. The ratios were plotted against pH and showed good agreement with the *in vitro* calibration curve. The pH_i_ of *S*. Typhi WT and the *ompR* null mutant were measured in MgM buffered to pH 7.2 or 5.6. Images were acquired and analyzed as described previously (Chakraborty et al., 2015, 2017).

### 2.5 pHi measurements using FLIM

For *in vitro* measurement, bacteria expressing mCherryTYG (Singh et al., 2025) were grown in LB pH 7.0 or 4.5 until OD_600_ ~1.0. Alternatively, an overnight culture of *S*. Typhi was used to inoculate MgM pH 7.2 at 0.2%. Bacteria were grown for 2 h before being shifted at pH 5.6. Bacterial cells were seeded onto poly-L-lysine-coated 8-well chambered slides, allowed to immobilize briefly, and rapidly imaged morphologically intact, clearly segmented bacteria as described below before the cells moved. For *in vivo* measurement, macrophages were infected by *S*. Typhi strains expressing mCherryTYG and maintained in phenol free RPMI. For intracellular measurements, only bacteria within clearly identifiable THP-1 cells and with contiguous mCherryTYG signal were analyzed (*n* ≈ 40–60 per condition) as described previously (Singh et al., 2025). Bacteria were identified in the mCherryTYG intensity channel using an automated intensity threshold, and their intracellular location was confirmed in the paired transmitted-light image; objects at the cell periphery whose intracellular position could not be assigned unambiguously were excluded. Individual bacteria were included in the lifetime analysis only if they were morphologically intact, clearly segmented from neighboring objects and accumulated sufficient photons for a stable biexponential fit. Because the intracellular burden is low at early time points, fields were sampled systematically across the whole chamber well rather than selected for high bacterial density: The reported *n* of 50 to 60 bacteria per condition was accumulated from three independent infections. The panels shown in Figure 3B are cropped single confocal planes selected for display and are not representative of the sampling density. Fluorescence lifetime imaging was performed using a TCS-SP8 3X system (Wetzlar, Germany) with a FLIM module. mCherryTYG was excited using a 20 MHz tunable white light laser at 561 nm, with emission collected at 580–720 nm via an in-built acousto-optical beam splitter. Photon detection (0.5–1 min) was carried out using a HyD detector. Images (256 × 256 pixels) were acquired at a scan speed of 400 Hz with a 1.0 Airy unit pinhole, using an HC PL APO CS2 100 × /1.40 oil immersion objective. The mCherryTYG lifetime histograms were analyzed using a biexponential decay model and deconvoluted with the IRF; lifetimes were then determined by global fitting in FLIMfit (Singh et al., 2025; Warren et al., 2013).

### 2.6 Super-resolution imaging

Super-resolution imaging (PALM) was performed as previously described (Foo et al., 2015; Liew et al., 2019) with modifications. Experiments were performed with *Salmonella* strains harboring *ssrB-*PAmCherry on the chromosome. For *in vitro* studies, bacteria were grown in LB at pH 7.0 or 4.5. Alternatively, infected macrophages were lysed by Triton 0.1%. bacteria were fixed by 1.5% PFA for 30 min and washed 3 times with PBS. The excess of PFA was quenched with an incubation of 15 min in 50 mM of Ammonium Chloride. Fixed bacteria were seeded onto glass bottom 8-well chambers (Sarstedt, Germany) coated with 0.1% of poly-L-Lysine (Sigma, USA). Chambers were previously cleaned with 3M KOH (Sigma, USA). Imaging was performed on a Nikon N-STORM Super-Resolution microscope. Activation of PAmCherry was performed by a 405 nm laser. Highly inclined and laminated optical sheet (HILO) illumination was done using 561 nm. To image the PAmCherry fusions, between 10,000–40,000 frames were acquired at an exposure time of 100 msec per frame. Super-resolution imaging data was analyzed using rapidSTORM (Wolter et al., 2012) as described previously (Liew et al., 2019). PAmCherry-tagged molecules in cells were quantified by generating localization-based images from PALM data using the LocAlization Microscopy Analyzer (LAMA; Malkusch and Heilemann, 2016). Cell boundaries were defined from brightfield images, and the number of molecules per cell was estimated from integrated intensity values. Since cell size varies with pH, molecule counts were normalized to cell area (molecules/μm^2^). Statistical analyses were then performed using GraphPad Prism 10.2.1.

### 2.7 Confocal imaging of P*sifA* activity

For *in vitro* confocal imaging of P*sifA* activity, overnight grown cultures of concerned *Salmonella* strains were sub-cultured (1:30 dilution) into 3 mL LB media adjusted to pH 7 and 4.5 and incubated at 37 °C until OD_600_ = 1. Bacteria were seeded and immobilized on agarose pads, preventing motility during acquisition. Alternatively, infected THP-1 were fixed with 4% PFA. All confocal imaging of P*sifA* activity in infected THP-1 cells was performed on fixed samples. Imaging was carried out using a Leica TCS SP8 STED 3X microscope (Leica Microsystems, Wetzlar, Germany) equipped with a tunable pulsed white light laser (470–670 nm) and a 405 nm UV diode laser for excitation at 561 nm and 405 nm. Two fluorescence channels were recorded: mTagBFP2 (excitation at 405 nm, emission collected between 440–470 nm) and mCherry (excitation at 561 nm, emission collected between 600–630 nm). The P*sifA* activity corresponded to the fluorescence intensity of mTagBFP2 divided by the constitutively expressed mCherry fluorescence for each individual bacterium as described previously (Fernandez et al., 2025). Ratio analysis was performed using the RatioPlus plugin from FIJI ImageJ (National Institutes of Health, Bethesda, MD, USA).

### 2.8 Statistical analysis

Statistical tests were performed in GraphPad Prism 10.2.1 (GraphPad Software, LLC, Boston, MA, USA) and are listed, with the corresponding *n*, in each figure legend. Comparisons between two groups used Student's *t*-test; comparisons among three or more groups used one-way ANOVA with correction for multiple comparisons. For replicate-level assays, *n* refers to the number of independent infections, each performed with four technical replicates, and each plotted point is the mean of one independent infection. For single-cell measurements, *n* refers to the number of individual bacteria or infected cells analyzed, pooled across the stated number of independent experiments, and each plotted point is one bacterium or one infected cell. For the LDH time course, absolute values were compared by two-way repeated-measures ANOVA with strain and time as factors and independent experiment as the blocking factor, and the 24 hpi comparison was additionally tested by ratio-paired *t*-test. Effect sizes for every quantitative comparison in this study, together with *n* and the test applied, are reported in Supplementary Table S2; the basis on which they are expressed, and the reason confidence intervals are not given, are stated in the legend to that table.

## 3 Results

### 3.1 *S*. Typhi net intracellular replication within the SCV of THP-1 macrophages is low

*S*. Typhi encounters immune cells during its infection of humans and its ability to survive within macrophages is key for its lifestyle. However, the pathogenesis of *S*. Typhi within human macrophages is poorly understood to date. It has been suggested that *S*. Typhi replication is much reduced in macrophages, unlike *S*. Typhimurium (Reuter et al., 2021). To characterize the pathogenesis of *S*. Typhi in THP-1 macrophages, we examined the intracellular burden of *S*. Typhi in THP-1 cells, using confocal microscopy. THP-1 cells were infected with *S*. Typhi or *S*. Typhimurium expressing mCherry. At 0.5 and 24 h post infection (hpi), samples were fixed and analyzed and the number of bacteria per infected cell was enumerated. Our results indicated that the intracellular burden at 24 hpi of *S*. Typhi H58, and of the attenuated strain *S*. Typhi Ty2, was low compared with *S*. Typhimurium (Figure 1A). The average number of *S*.Typhi H58 was 2 bacteria/cell compared to 5 bacteria/cell for Ty2 and 8 bacteria/cell for *S*. Typhimurium. Moreover, the maximum bacteria per infected THP-1 was around 20 for *S*. Typhi strains but reached more than 50 for *S*. Typhimurium. Effect sizes, *n* and the statistical test for this and all subsequent comparisons are reported in Supplementary Table S2. A similar observation was reported for the *S*. Typhimurium strain NCTC12023 in comparison to *S*. Typhi strain 120130191 (Reuter et al., 2021). Some differences among *S*. Typhi strains were noted, as we observed a lower intracellular burden for H58 than for Ty2.

We next examined whether bacteria were indeed in vacuoles, by examining their colocalization with LAMP-1, a common marker for the SCV. Immunostaining of LAMP-1 was performed on fixed samples following infection. Because LAMP-1 staining was distributed throughout the macrophage cytoplasm, we scored a bacterium as LAMP-1-positive only when LAMP-1 signal enclosed the bacterial outline in a continuous or near-continuous ring in the plane of best focus, rather than scoring simple pixel overlap. Our images revealed that most of the *S*. Typhi bacteria were surrounded by or co-localized with LAMP-1, forming what appeared to be individual bacteria-containing vacuoles (Figure 1B). Taken together, these results suggested that during infection, *S*. Typhi largely resided in vacuoles inside human macrophages, where its net replication was low.

### 3.2 An *ssrB* deletion in *S*. Typhi differs phenotypically from *S*. Typhimurium

The gentamicin protection assay is a standard assay for determining uptake and survival of intracellular bacteria (see Section 2). We performed gentamicin protection assays with our H58 *S*. Typhi strain. As previously described with *S*. Typhi strain ISP1820 (Forest et al., 2010), deletion of *ssrB* did not appear to have a distinct phenotype compared to the parental WT strain (Figures 2A, B). We anticipated that *ssrB* deletion would drastically reduce the number of intracellular bacteria, as we observed with *S*. Typhimurium (Shetty and Kenney, 2023). In parallel, prevention of synthesis and assembly of the SPI-1 T3SS by deletion *of prgHIJ* had no effect on the bacteria survival, but SPI-1 deletion in the *ssrB* null strain decreased bacterial survival by 60% compared to the WT (Figure 2B). This result indicated that SsrB was required for intracellular survival in the absence of the SPI-1 injectosome. This result is consistent with those of Hamblin et al. who suggested that SPI-2 effectors were capable of being secreted through the SPI-1 needle and *vice-versa* (Hamblin et al., 2023). As the gentamicin assay did not provide complete understanding, we next investigated intracellular burden by confocal microscopy. THP-1 cells were infected with WT or *ssrB* null strains of *S*. Typhi H58 or *S*. Typhimurium 14028s expressing mCherry. At 0.5 and 24 hpi, samples were fixed and the number of intracellular bacteria per infected macrophages were enumerated. As expected, *ssrB* was essential for *S*. Typhimurium survival in macrophages (Feng et al., 2003; Walthers et al., 2007) as its deletion decreased the number of intracellular bacteria. The maximum number of bacteria per infected THP-1 cell was more than 70 for the WT strain, but only 22 for the *ssrB* null strain (Figure 2C). Surprisingly, and in contrast to *S*. Typhimurium, deletion of *ssrB* in *S*. Typhi H58 led to a small, but significant increase in intracellular burden at 24 hpi (Figures 2C, D).

**Figure 2:**
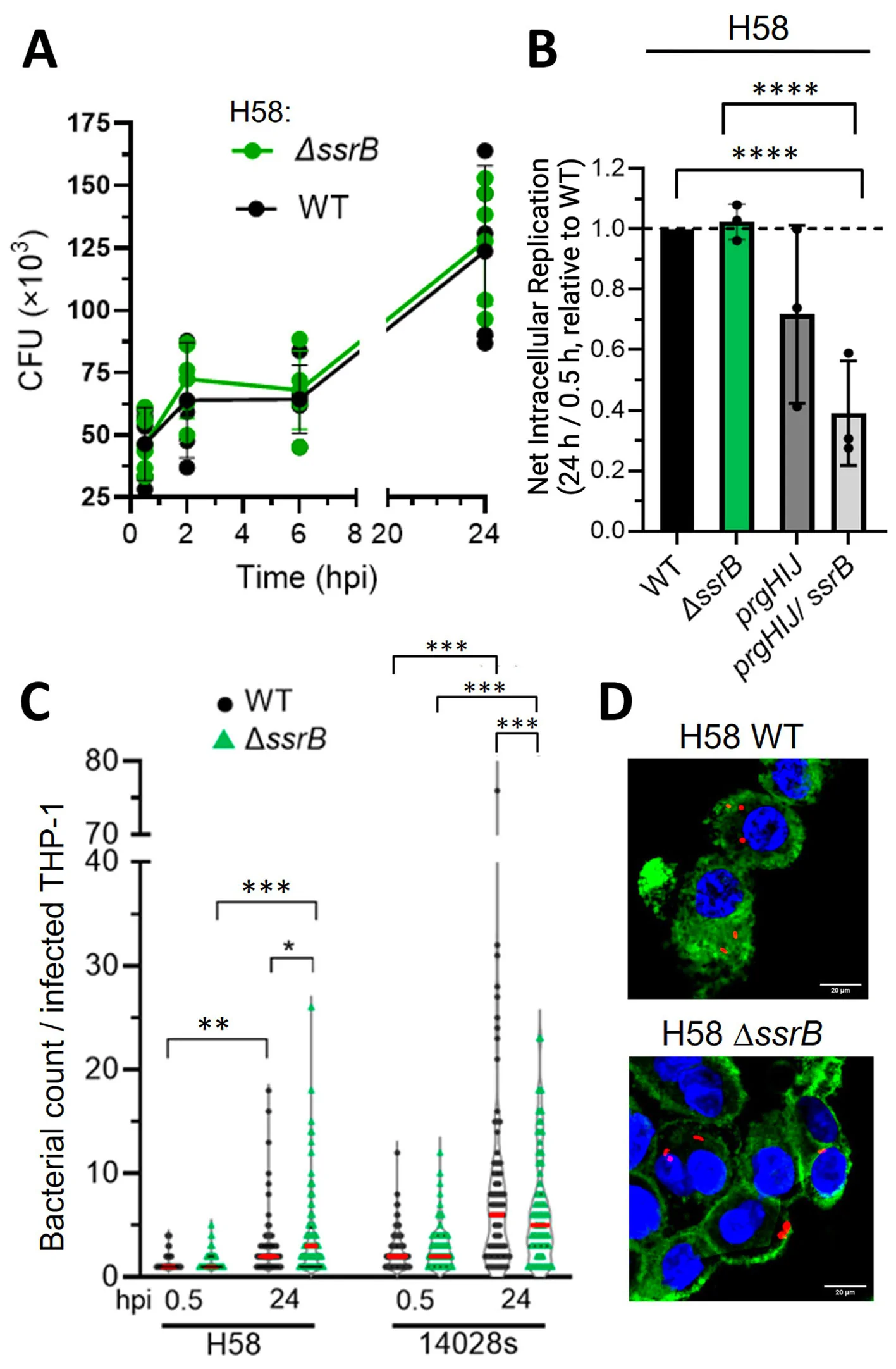
Bacterial enumeration assays present an ambiguous role for SsrB in *S*. Typhi pathogenesis. THP-1 cells were infected with *S*Ty H58. The bacterial burden was estimated at each time point post infection by macrophage lysis and enumeration after agar plating. **(A)** Bacterial load at indicated time points. **(B)** The ratios of CFU at 24 hpi/30 min pi obtained for the null mutants were normalized to the parental strain (WT). Data were from 5 independent experiments, each with four technical replicates for each strain; each point is the mean of one independent experiment. Error bars indicate standard deviation (SD). ****p < 0.0001 (one-way ANOVA); brackets indicate the comparisons made. **(C)** Deletion of *ssrB* results in different apparent phenotypes for *S*Ty and *S*Tm. The number of bacteria per infected THP-1 macrophage was enumerated by confocal microscopy at 0.5 and 24 hpi. The strains were *S*Ty H58 and *S*Tm 14028s (black dots) as well as the corresponding *ssrB* null mutants (green triangles). Each dot or triangle represents one infected THP-1 cell (*n* = 100). *p < 0.05, **p < 0.01 and ***p < 0.001 (One-way ANOVA). **(D)** Representative confocal images of THP-1 cells infected with *S*Ty H58 pFPV-mCherry, at 24 hpi. Cells were fixed with 4% PFA and membranes were labeled using MemGlow. Red, *Salmonella*; Blue, DAPI; Green, plasma membrane. Images shown in this figure were selected from datasets used for the corresponding quantification. Scale bar: 2 μm.

### 3.3 The *S*. Typhi cytosol acidifies in response to the acidic environment of the SCV

In previous studies from our laboratory, we determined that the cytosol of *S*. Typhimurium was acidified in response to low external pH *in vitro* and during survival in the acidic vacuole inside host cells (Chakraborty et al., 2015, 2017). This process was dependent on EnvZ/OmpR two-component signaling and involved repression of the *cadC/BA* lysine decarboxylation system to prevent neutralization of the bacterial cytoplasm (Chakraborty et al., 2015, 2017; Chakraborty and Kenney, 2018). Intracellular acidification was crucial for *S*. Typhimurium, as it triggered expression of SPI-2 genes through a pH-dependent switch in the SsrB response regulator (Liew et al., 2019; Shetty and Kenney, 2023). Because there did not appear to be a difference in the number of intracellular bacteria in the absence or presence of SsrB in *S*. Typhi (Figure 2), we were therefore interested in determining the *S*. Typhi response to acid stress. We first monitored bacterial internal pH (pH_i_) using the dye BCECF, whose fluorescence emission is pH-dependent (Supplementary Figure S1A). Experiments performed both *in vitro* and in THP-1 cells with the H58 strain highlight that bacteria indeed acidified in response to acidic environments (Supplementary Figure S1B). Because OmpR was important for bacterial acidification in *S*. Typhimurium, we investigated its role in *S*. Typhi. The *ompR* null strain was still able to acidify, although its pHi before the acidic shift was higher than that of the *S*. Typhi WT strain (7.30 vs. 6.81, Supplementary Figure S1B). OmpR is therefore not required for cytoplasmic acidification in *S*. Typhi H58, in contrast to *S*. Typhimurium. The labeling of STy bacteria was not stable upon cell infection and did not allow long-term measurements. Therefore, we shifted to a variant of the mCherry protein, mCherryTYG, which substitutes threonine for methionine 66 and exhibits a fluorescence lifetime that is pH-dependent (Haynes et al., 2019; Singh et al., 2025). Using the mCherryTYG probe, we demonstrated that the *S*. Typhi H58 cytosol acidified from pH 7 to 6.6 (in MgM pH 5.6) and to 6.3 (in LB pH 4.5) when bacteria were shifted from neutral to acidic pH (Figure 3A). We noticed an increase of heterogeneity in comparison to what we previously observed for *S*. Typhimurium (Singh et al., 2025). Furthermore, we were able to track the bacterial pH_i_ inside THP-1 macrophages after infection with *S*. Typhi expressing the mCherryTYG probe (Figures 3A, B). After 24 hpi, the bacterial cytosol shifted from a starting pH of ~6.8 to pH 5.7. This value was very close to that reported for *S*. Typhimurium (pH = 5.6) (Chakraborty et al., 2015), although the *S*Tm bacteria reached this value quite rapidly (within 30 min in RAW 264.7 macrophages). Further, the response of *S*Tm after 24 h was relatively homogenous and the entire intracellular population was acidified. We were unable to determine the subcellular location of individual bacteria in the same experiment in which pHi was measured, because a fluorescent LAMP-1 fusion could not be stably expressed in the THP-1 line. Vacuolar residence of the bacteria analyzed by FLIM is therefore an assumption of the analysis that follows, and not demonstrated. Two observations make the assumption reasonable. First, LAMP-1 immunostaining of fixed infected cells showed *S*. Typhi associated with LAMP-1-positive compartments at 24 hpi (Figure 1B); this staining was qualitative, was not quantified, and was not performed at earlier time points. Second, the intracellular population was uniformly acidified at every time point examined after infection, with no near-neutral subpopulation (Figure 3A), and cytosolic *Salmonella* maintain a near-neutral pHi (Singh et al., 2025). Limited cytosolic access has also been reported for typhoidal serovars in human macrophages (Reuter et al., 2021). None of this excludes the presence of cytosolic bacteria among those analyzed here, and the pHi values reported below should be read as those of the acidified, probe-retaining population rather than of the entire intracellular population. A direct test would require simultaneous measurement of pHi and compartment identity in living cells.

**Figure 3:**
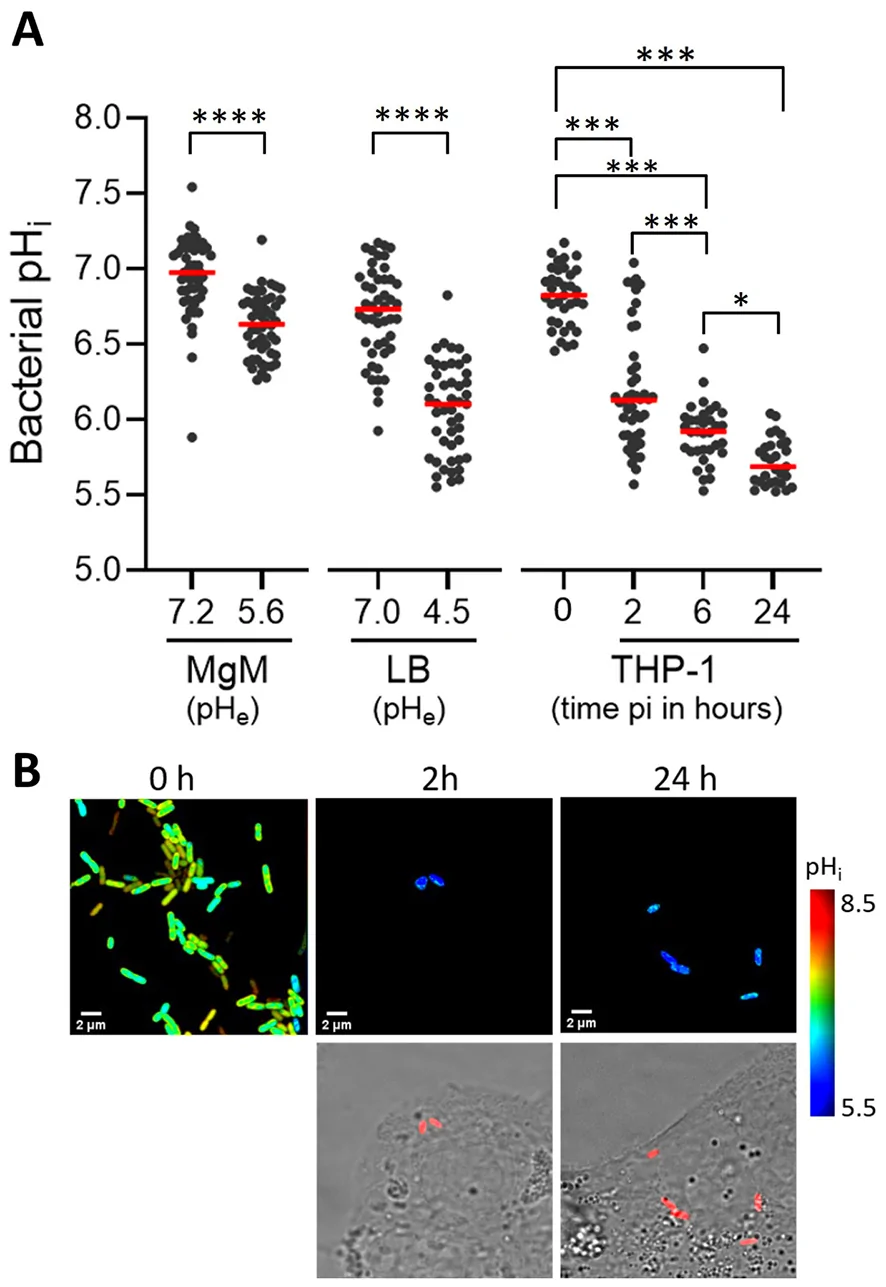
*S*. Typhi acidifies its cytosol in response to the acidic vacuolar environment in THP-1 cells. *S*. Typhi H58 expressing pFPV-mCherryTYG were grown *in vitro* in MgM pH 7.2 or LB pH 7 before being shifted to MgM pH 5.6 or LB pH 4.5 for 1 h. Alternatively, bacteria were used to infect THP-1 cells. At the indicated time point, the bacterial pH_i_ was determined using the mCherryTYG fluorescent lifetime, measured by confocal microscopy (see Section 2). **(A)** Dots represent lifetime values of individual bacteria, which were converted to pHi (Singh et al., 2025); For each condition, *n* = 50–60 bacteria from three independent experiments. **p<0.01, ****p < 0.0001 (Student's *t*-test for *in vitro* and One-way ANOVA for *in vivo* determinations). **(B)** Representative images of infected THP-1 cells. Red, *Salmonella* containing pFPV-mCherryTYG are color-coded from blue to red, depending on the intracellular pH_i_ as presented in the legend. Images shown were selected from datasets used for the corresponding quantification. Scale bar: 2 μm.

Lastly, our measurements in the *ompR* null strain highlighted an acidification, although the starting pH_i_ was higher at the time of cell entry, resulting in a higher intracellular pH over the same time course (Supplementary Figure S1C). Taken together, our results highlight that cytosolic acidification is an important feature in the infection of host cells by *S*. Typhi, as we previously observed with *S*. Typhimurium.

### 3.4 Acidification drives SsrB-dependent *sifA* transcription, which is lower in *S*. Typhi than in *S*. Typhimurium

In *S*. Typhimurium, bacterial acidification is an essential step for bacterial survival within host cells and involves activation of SsrB and its regulon (Chakraborty et al., 2017, 2015). Since *S*. Typhi acidified in response to acidic stress (Figure 3), it was of interest to verify that SsrB activity was influenced by pH_i_ as well. As a single-cell indicator of SsrB activity under acidic conditions, we employed P_*sifA*_. This reports on one directly SsrB-activated, H-NS-silenced SPI-2 promoter that has been characterized biochemically (Walthers et al., 2007, 2011). Activation of P_*sifA*_ was monitored using a transcriptional fusion of the promoter to the gene encoding the BFP protein. For each tested condition, BFP fluorescence was normalized to constitutive mCherry fluorescence. *In vitro*, the WT strain exhibited an approximately 2-fold increase in *P*_*sifA*_ activity at acidic pH compared to neutral pH (Figure 4). Acid induction of P_*sifA*_ was not apparent in the *ssrB* null mutant, indicating that expression of *sifA* was completely dependent on SsrB in *S*. Typhi, as it was in *S*. Typhimurium (Walthers et al., 2007). Transcriptional activation was heterogeneous among intracellular bacteria (Figure 4A), as we also observed when counting SsrB molecules (Figure 5). Consistent with a requirement for acidification, a strain overproducing the *cadC/BA* lysine decarboxylation system, which does not acidify its cytoplasm (Chakraborty et al., 2015), showed P_*sifA*_ activity at acid pH that was lower even than at neutral pH (Figure 4A). We note that *cadC/BA* overproduction also affects amino acid metabolism and membrane physiology, so this result is consistent with a strict requirement for cytoplasmic acidification. An *ompR* null strain would not provide a cleaner test in this serovar, since *S*. Typhi H58 lacking *ompR* still acidifies (Supplementary Figure S1) and OmpR directly activates *ssrA/B* transcription (Feng et al., 2003). Previously, Reuter et al., reported that SPI-2 activation was lower in *S*. Typhi compared to *S*. Typhimurium, and effector secretion was also reduced (Reuter et al., 2021). This phenotype was reproduced in the H58 strain (Fernandez et al., 2025) and as shown in Figure 4B. Monitoring of P_*sifA*_ activity by confocal microscopy determined that *sifA* activation in *S*. Typhi was reduced compared to *S*. Typhimurium. Only ~70% of *S*. Typhi bacteria activated P_*sifA*_ *in vivo* at 24 hpi compared to >90% for *S*. Typhimurium, and the level of activation per cell was also ~50% lower on average. Because these measurements report on a single promoter, and because SsrB-dependent promoters differ in SsrB-binding affinity and in the extent of H-NS silencing, we do not extrapolate from P_*sifA*_ to the SPI-2 regulon as a whole.

**Figure 4:**
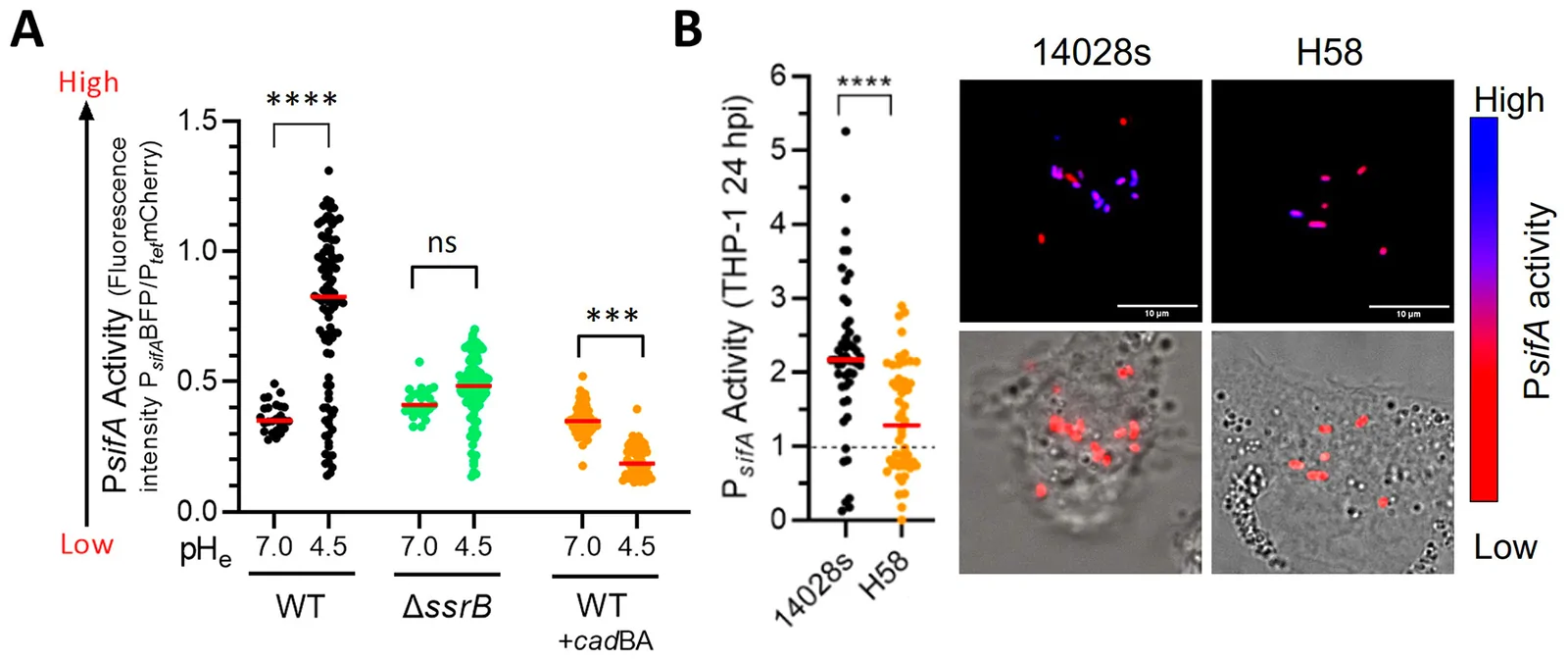
*S*. Typhi acidification triggers lower *sifA* activation by SsrB compared to *S*. Typhimurium. The P*sifA* reporter was a low copy plasmid, expressing mTagBFP under the control of P*sifA*, as well as constitutive mCherry under the control of P*tet*. Two sets of images were acquired by confocal microscopy corresponding to the excitation and emission of both fluorophores, the BFP fluorescence was normalized to the mCherry fluorescence for each individual bacterium. **(A)** *S*Ty H58 strains were grown *in vitro* under neutral or acidic (pH 4.5) conditions and analyzed by confocal microscopy imaging. The dots on the graph represent the BFP/mCherry ratio for each bacterium. Activation of P*sifA* is *ssrB*-dependent. Bacteria expressing *cadC/BA* from a plasmid were unable to acidify (Chakraborty et al., 2015) and failed to activate P_*sifA*_. *n* = ~50 bacteria; ***p < 0.001, ****p < 0.0001 (One-way ANOVA). **(B)** THP-1 cells were infected with *S*Tm 14028s or *S*Ty H58. Samples were analyzed at 24 hpi by confocal microscopy. Right Panel: Each dot represents an individual bacterium; *n* = 50 bacteria pooled across three independent infections. ****p < 0.0001, Student's *t*-test. Left panel: Representative pictures, images are color-coded for P*sifA* activity. Images shown in this figure were selected from datasets used for the corresponding quantification. Scale bar: 10 μm.

**Figure 5:**
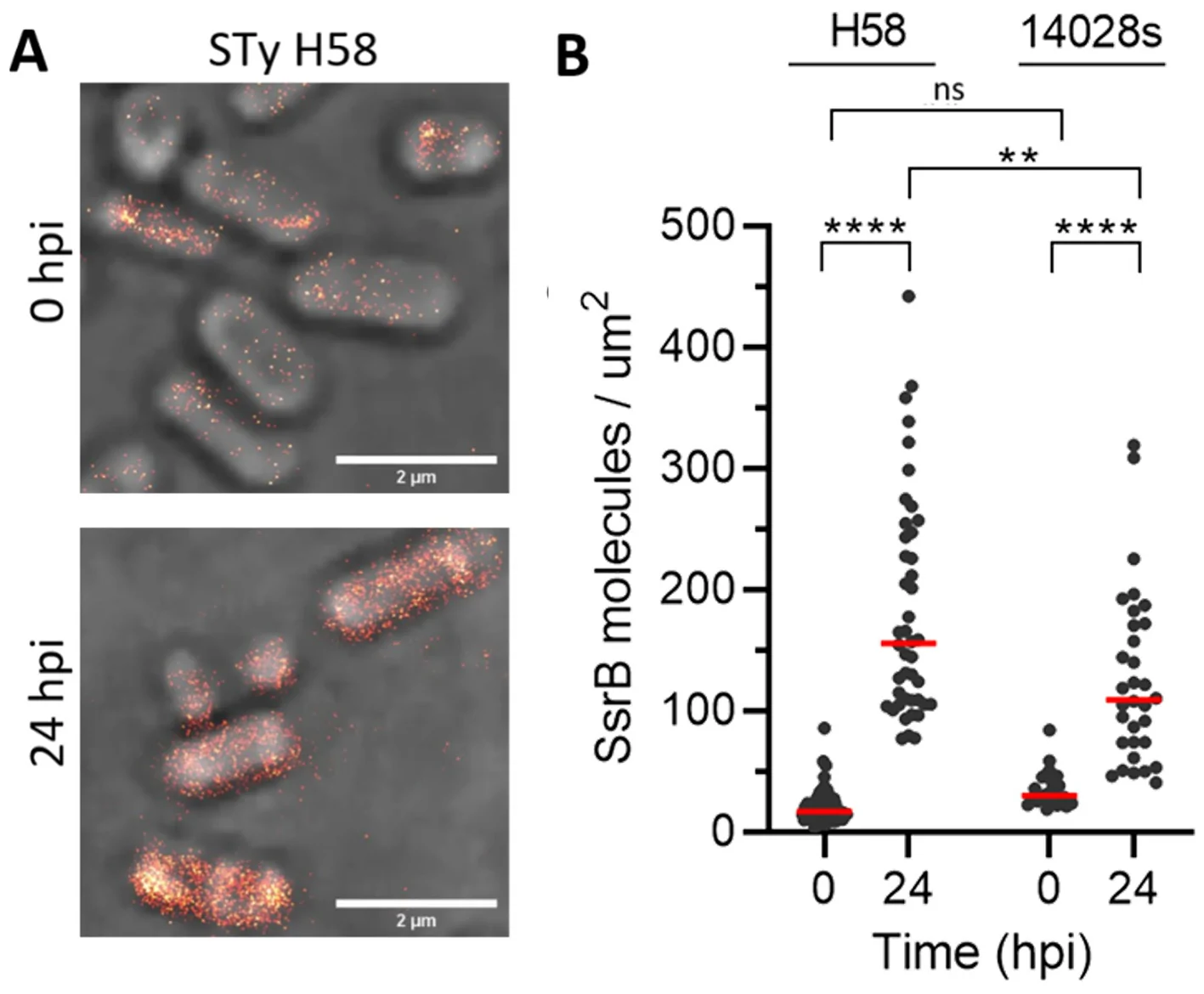
In *S*. Typhi, SsrB is up-regulated in THP-1 macrophages compared to *S*. Typhimurium. Quantification of SsrB molecules in *Salmonella* in infected THP-1. THP-1 cells were infected with *S*Ty H58 or *S*Tm 14028s, expressing a translational fusion of *ssrB* with photoactivable PAmCherry Δ*ssrB*::*kan attB:*: PAmCherry-ssrB). At 24 hpi, macrophages were lysed and bacteria were fixed and prepared for microscopy. Alternatively, bacteria were fixed and analyzed from the infection inoculum (0 hpi). **(A)** Representative PALM-Brightfield overlay images of *Salmonella* expressing PAmCherry-SsrB (red dots). Scale bar: 2 μm. **(B)** Quantification of SsrB molecules in both serovars. Each dot represents data from a single bacterium (*n* = 45–50 bacteria per sample from three independent experiments). Images shown in this figure were selected from datasets used for the corresponding quantification. **p < 0.01, ****p < 0.0001, One-way ANOVA.

### 3.5 SsrB is activated upon acidic stress in THP-1 macrophages

As presented in Figure 3, the *S*. Typhi cytosol was acidified to the same extent as we observed in *S*. Typhimurium (Chakraborty et al., 2015, 2017). We next determined whether the lower expression of P*sifA* observed in Figure 4 was due to a lower production of SsrB by *S*. Typhi in comparison to *S*. Typhimurium. We analyzed the number of SsrB molecules in bacteria using photoactivated localization microscopy (PALM), PALM-based counting methods and super-resolution imaging, as we previously described (Liew et al., 2019). From the images, it is apparent that SsrB was drastically up-regulated upon *S*. Typhi entry into THP-1 cells (~10-fold, Figures 5A, B). Surprisingly, the number of SsrB per bacteria was significantly higher for *S*. Typhi than for *S*. Typhimurium (an average of 160 molecules for *S*. Typhi compared to 125 for *S*Tm; Figure 5B). Therefore the differential activation of *sifA* between *S*. Typhi and *S*. Typhimurium (Figure 4) was not due to a lack of SsrB production, but rather to a difference in the activity of SsrB at this promoter between the two serovars. We previously reported that substantial differences in the redox environment of the two strains exists and this higher redox in *S*. Typhi modifies SsrB, reducing its activity (Fernandez et al., 2025). We also determined the number of SsrB molecules *in vitro* (in LB) in *S*. Typhi. The number of SsrB molecules/bacteria in the exponential growth phase slightly increased from 32 to 41 when bacteria were grown under acidic conditions compared to neutral pH (Supplementary Figure S2). The increase in SsrB expression was also growth phase-dependent, as there were more SsrB molecules in the stationary phase compared to the exponential phase (an average of 55 vs. 41, respectively). We noted the drastic difference in SsrB production in THP-1 cells vs. acidic LB (>three-fold). Together these results demonstrate that activation of SsrB production in *S*. Typhi was triggered by acidic conditions and that the SCV is a unique environment that favors an increase in SsrB production.

### 3.6 SsrB limits host cytotoxicity and restricts net intracellular replication, with different requirements for phosphorylation and acid sensing

Our results thus far suggested that SsrB restricts rather than promotes net intracellular replication of *S*. Typhi in macrophages. Ensemble-based assays such as the gentamicin protection assay followed by bacterial enumeration have limitations. In particular, they overlook the host cell death induced by the pathogen, potentially leading to an underestimation of the bacterial load. To further examine the *in vivo* phenotype, we investigated *S*. Typhi cytotoxic effects on THP-1 cells by measuring a time course of LDH release (Figure 6A). At 24 hpi, the *ssrB* null strain released more LDH than the WT strain. In the unnormalized time course, the difference was of the expected magnitude but was not resolved statistically with three independent infections (Figure 6A); in a larger set of infections in which each mutant was normalized to its paired WT control, the *ssrB* null strain was approximately 65% more cytotoxic than the WT (Figure 7A). This result highlights that SsrB plays an important role in reducing *S*. Typhi cytotoxicity in human macrophages.

**Figure 6:**
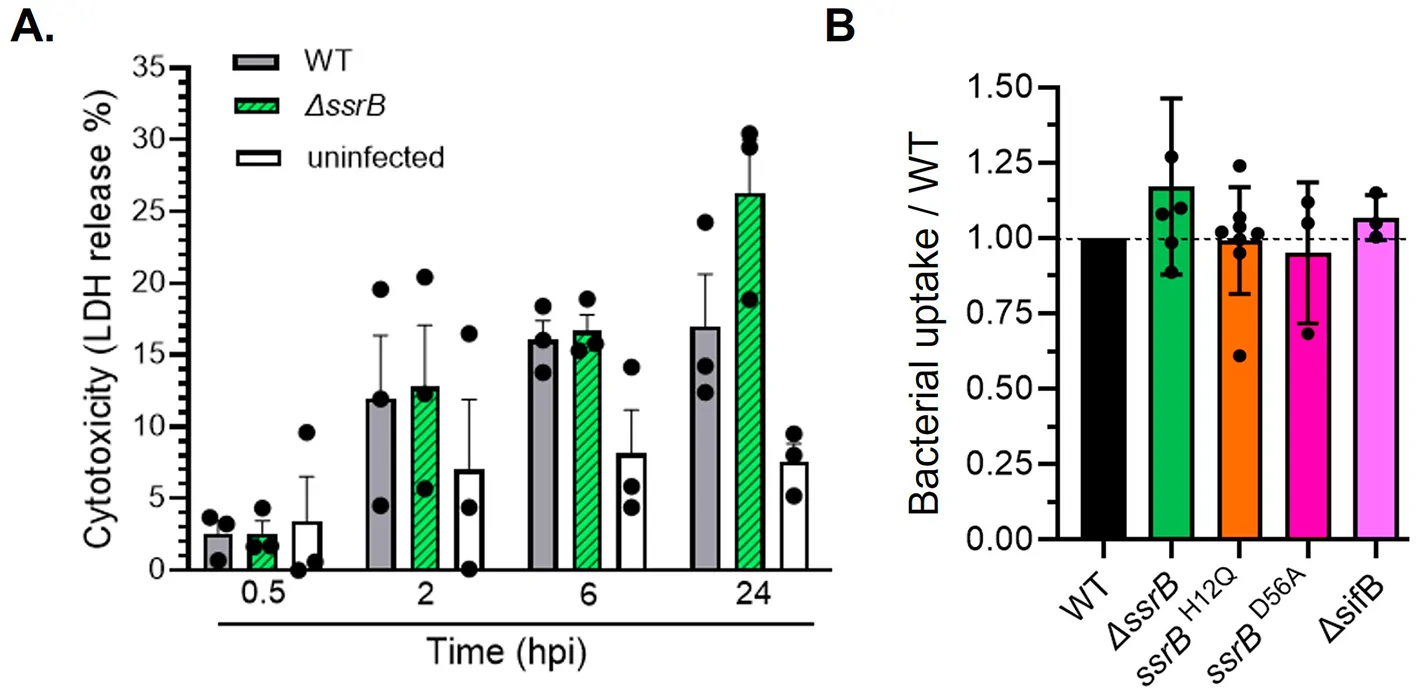
SsrB reduces the bacterial cytotoxicity in THP-1 cells. **(A)** Cytotoxicity of *S*Ty H58 strains toward THP-1 cells was measured as LDH release and expressed as a percentage of a maximum-lysis control. Measurements were performed at 0.5, 2, 6 and 24 hpi. Data are from three independent infections, each with four technical replicates; each point is the mean of one independent infection. Absolute values were compared by two-way repeated-measures ANOVA (strain and time), with an independent experiment as the blocking factor [*p* = 0.087 for WT vs. the *ssrB* null strain at 24 hpi]. Mean absolute cytotoxicity at 24 hpi was 17% for WT and 26% for the *ssrB* null strain. The time-course dataset, which used unnormalized values and *n* = 3, was not powered to resolve this difference; the same comparison performed on a larger set of independent infections and normalized within each experiment to the paired WT control is shown in Figure 7A. **(B)** THP-1 cells were infected with *S*Ty H58 strains. Uptake was estimated at 30 min post infection by macrophage lysis and enumeration after agar plating. Data are from four independent infections with four technical replicates per strain and are presented as mean ± SD; each point is the mean of one independent infection. Differences between strains were not significant (one-way ANOVA).

**Figure 7:**
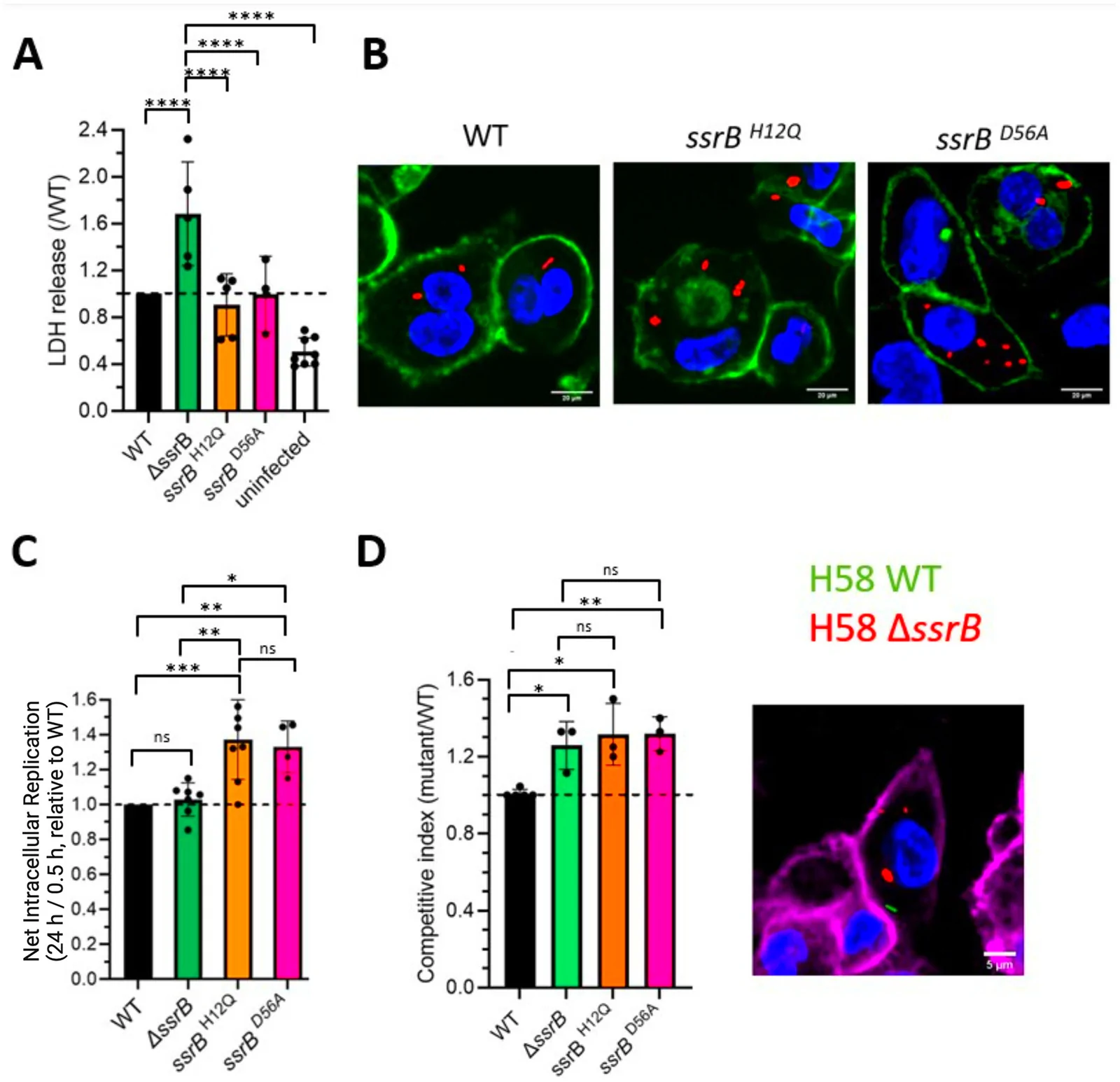
SsrB and SsrB~P reduce the cytotoxicity and *S*. Typhi net intracellular replication during infection of THP-1 macrophages. **(A)** The cytotoxic effect of *S*Ty H58 strains on THP-1 cells was determined by measuring LDH release. LDH activity in the THP-1 supernatant at 24 hpi was normalized to WT. Five independent experiments were performed with four replicates. Measurements were performed at 24 hpi. ****p < 0.0001 (One-way ANOVA). **(B)** Representative images of infected THP-1 cells at 24 hpi. Samples were fixed and analyzed by confocal microscopy, Red, *Salmonella*, Blue, DAPI, green, THP-1 membranes (MemGlow). Images shown in this figure were selected from datasets used for the corresponding quantification. Scale bar: 20 μm. **(C)** The bacterial load of *S*Ty H58 strains was assessed by plating on LB agar after 30 min (Uptake) and 24 hpi. The ratios of the bacterial load at 24 hpi/ 30 min pi obtained for the mutants were normalized to the WT. Data are from three independent infections, each with four technical replicates, and are presented as mean ± SD; each point is the mean of one independent infection.*p < 0.05 **p < 0.01, ***p < 0.001, One-way ANOVA. **(D)** THP-1 cells were co-infected with *S*Ty H58 mutant strains expressing pFPV-mCherry, and H58 WT expressing pFPV25-gfp. At 24 hpi, the number of mCherry and GFP bacteria per individual co-infected THP-1 cell was determined by confocal imaging. Data are from three independent infections, each with four technical replicates and 50 co-infected THP-1 cells analyzed per experiment; each point is the competitive index of one independent infection. Left panel: Competitive index (mutant/WT bacteria). *p < 0.05, **p < 0.01, One-way ANOVA. At 30 min post-infection, competitive indices were not significantly different from 1. Right panel: Representative confocal image of a THP-1 cell co-infected with H58 WT (green) and *ssrB* null (red). Nuclei are stained with DAPI (blue), and THP-1 membranes are labeled with MemGlow (magenta). Scale bar: 5 μm.

Our analysis included two chromosomal SsrB point mutants, H12Q and D56A. The H12Q substitution prevents the increase in DNA-binding affinity that wild-type SsrB exhibits at acid pH, while leaving DNA-binding affinity and transcriptional activity at neutral pH essentially unchanged and the protein phosphorylation-competent (Shetty and Kenney, 2023); D56A removes the phosphoacceptor aspartate and thus prevents phosphorylation by the SsrA kinase (Feng et al., 2004). Steady-state levels of the two mutant proteins were not determined in *S*. Typhi H58 (see Section 4). Neither mutant displayed an invasion defect (Figure 6B) and both mutants had the same level of LDH release compared to WT (Figure 7A). The significance of this result is that whatever SsrB regulates in order to limit host cytotoxicity, which is the phenotype measured in this assay, requires neither phosphorylation at D56 nor the H12-dependent increase in DNA-binding affinity. A repressive activity of unphosphorylated SsrB would be consistent with this pattern, although it is not the only explanation (see Section 4). In parallel, the mutants exhibited a higher net intracellular replication rate compared to the WT and the *ssrB* null mutant when investigated by both confocal microscopy (representative images, Figure 7B; quantification, Supplementary Figure S3) or a gentamicin protection assay (H12Q, +37%; D56A, +32%; Figure 7C). We noted a higher number of bacteria per cell in the SsrB^D56A^ or SsrB^H12Q^ mutants compared to the *ssrB* null strain (Supplementary Figure S3). This result further emphasizes that the higher cytotoxicity effect of the *ssrB* null strain biases the analysis of net intracellular replication, even by single cell microscopy analysis.

Finally, to remove the cytotoxicity bias, we performed a competitive infection where we infected THP-1 cells with equal numbers of WT and mutant bacteria. We then compared the number of mCherry-expressing mutant bacteria in co-infected THP-1 cells with the number of GFP-expressing WT, after 24 hpi (Figure 7D). The number of mutant bacteria per infected THP-1 cell was higher than the number of WT bacteria (~+30% competitive index). The competitive index at 0.5 hpi was equal to 1, removing the possibility of an invasion defect bias. Thus, SsrB contributes to two infection phenotypes with different genetic requirements: it limits cytotoxicity toward the host cell in a manner requiring neither phosphorylation at D56 nor H12-dependent acid sensing, and it restricts net intracellular replication in a manner requiring both activities. The D56A and H12Q alleles alter net intracellular replication without altering cytotoxicity, whereas the *ssrB* null strain alters cytotoxicity; the two phenotypes therefore have different upstream requirements. Whether they are executed by shared downstream targets was not determined here. We note that the *ssrB* null strain also exhibited a small increase in intracellular burden by microscopy (Figure 2C), which the elevated cytotoxicity of that strain was likely to influence.

### 3.7 Activation of *sifB* by SsrB reduces *S*. Typhi net intracellular replication in THP-1 cells

The ability of SsrB to respond to acid stress requires H12 and phosphorylation at D56 in order to reduce net intracellular replication in THP-1 cells. It was of interest to identify targets of SsrB regulation that were responsible for restricting net intracellular replication inside host cells. We examined well-characterized members of the SsrB regulon, i.e., the SPI-2 regulon and SPI-2 co-regulated genes. Recent studies suggested that in the well-studied vaccine strain Ty2, deletions of *steC* and *sifB* resulted in higher growth in THP-1 cells (Hamblin et al., 2023), although the results with *sifB* were not significant. A transcriptomic study of strain ISP18 reported that activation of *sifB* in THP-1 cells was five times greater than the inoculum after 24 hpi, whereas *sifA* expression only increased by a factor of two (Faucher et al., 2006); *steC* was not activated. Because of our interest in SsrB-activated genes, we focused on *sifB*. Activation of *sifB* by SsrB was greater than *sifA* (Walthers et al., 2011). Although it was identified years ago, little is known about the role of *sifB* in *Salmonella* pathogenesis. Its deletion does not impact the ability of *S*. Typhimurium to produce *Salmonella*-induced filaments (SIFs) in the HeLa cell infection model (Rajashekar et al., 2014). We deleted *sifB* and then used the *sifB* null strain to compare with the WT parent. Net intracellular replication of the *sifB* null strain was 8% higher than that of the WT strain by gentamicin protection assay (Figure 8A). The increase was relatively minor compared to the SsrB^H12Q^ and SsrB^D56A^ activation mutants, suggesting that *sifB* was not the only SsrB target involved in the regulation of net intracellular replication *in vivo*. Nevertheless, microscopy analysis revealed a higher intracellular burden for the *sifB* null strain than for the WT, both in the context of a simple infection (Figure 8B) or by co-infection (i.e., 40% more *sifB* null mutant bacteria than WT, Figure 8C). Finally, expressing *sifB* from a constitutive promoter in the *ssrB*^*D*56*A*^ and ssrB^*H*12*Q*^ strains reduced their net intracellular replication (Figure 8D). The reduction was significant for the D56A strain (from 1.31 to 0.83 relative to the WT), whereas in the H12Q strain the change was in the same direction but did not reach significance (from 1.38 to 1.13). The complemented H12Q strain was, however, no longer significantly different from the WT. Taken together, these results identify *sifB* as one of the targets through which SsrB activation reduces net intracellular replication of *S*. Typhi. The *sifB* deletion and constitutive complementation experiments in Figure 8 provide a second, independent line of evidence for SsrB-dependent output in *S*. Typhi. This evidence is genetic rather than transcriptional and does not depend on the P_*sifA*_ reporter.

**Figure 8:**
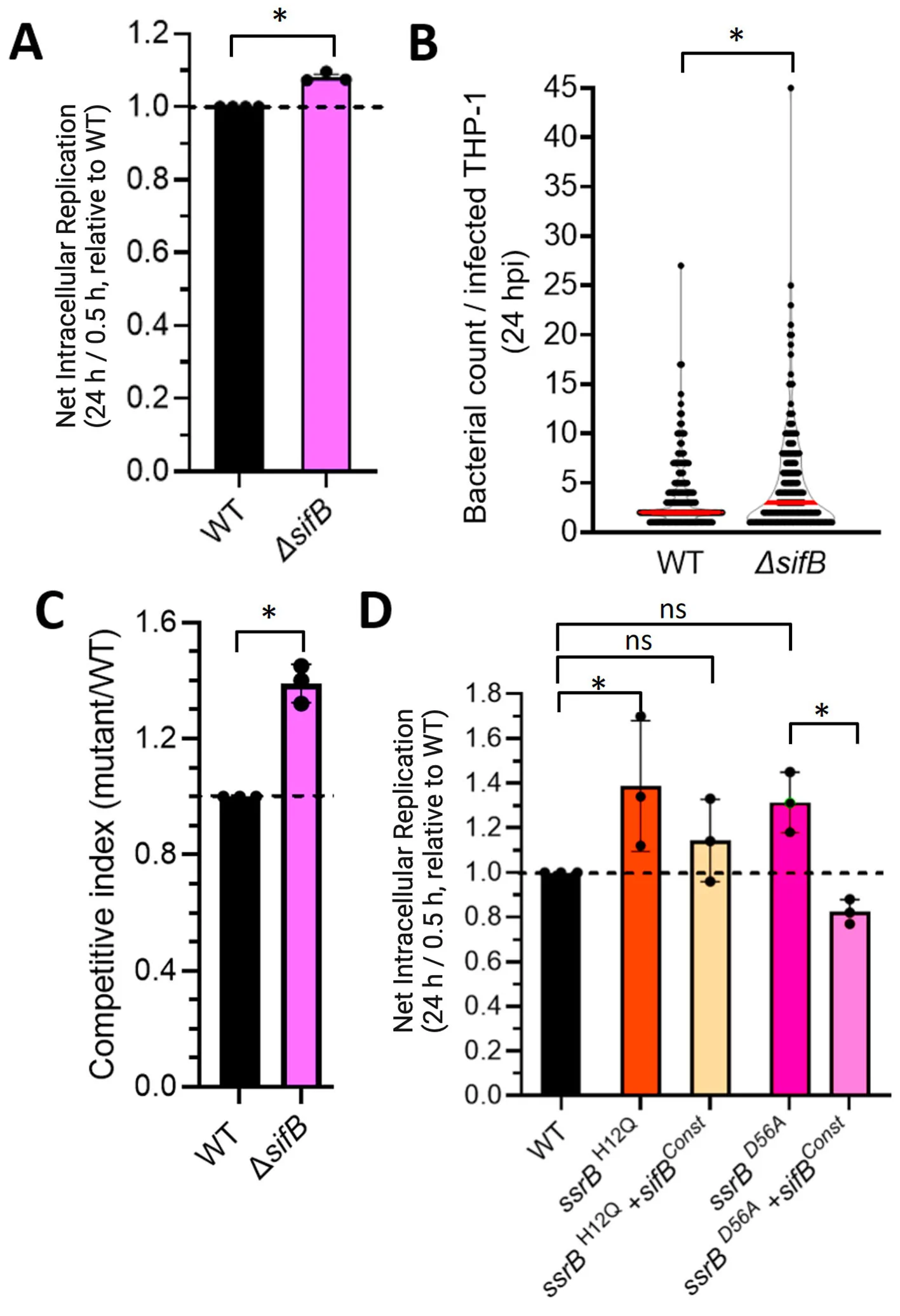
Induction of *sifB* reduces *S*. Typhi net intracellular replication in THP-1 macrophages. **(A)** The bacterial load of *S*Ty H58 strains was assessed by plating on LB agar after 30 min (Uptake) and 24 hpi. The ratios of the bacterial load at 24 hpi/ 30 min pi obtained for the mutants were normalized to WT. Data are from three independent infections, each with four technical replicates, and are presented as mean ± SD. *p < 0.05, Student's *t*-test. **(B)** THP-1 cells were infected with an mCherry-expressing strain. At 24 h post-infection (hpi), the number of bacteria per infected THP-1 cell was enumerated (*n* = 100 infected cells per condition, from three independent infections); each point is one infected cell. *p < 0.05, Student's *t*-test. **(C)** THP-1 cells were co-infected with *S*Ty H58 mutant strains expressing pFPV-mCherry, and H58 WT expressing pFPV25-gfp. At 24 hpi, the number of mCherry and GFP bacteria per individual co-infected THP-1 cell was determined by confocal microscopy imaging. Data represent competitive indices from experiments analyzing 50 THP-1 cells per experiment (mutant/WT bacteria). ***p < 0.001, Student's *t*-test. Competitive indices at 30 min post-infection were not significantly different from 1. **(D)** Gentamicin protection assay of the *ssrB* mutant (H12Q and D56A) *S*. Typhi H58 with or without constitutive plasmid-driven expression of *sifB*. Data are from three independent infections, each with four technical replicates. *p < 0.05; ns, not significant; one-way ANOVA.

## 4 Discussion

Unlike *S*. Typhimurium, the pathogenicity of *S*. Typhi remains far less understood, especially regarding its lifestyle within the host cell. Herein, we analyzed *S*. Typhi H58 pathogenesis in THP-1 human macrophages with a specific focus on SsrB. Our results reveal additional roles for this regulator compared to *S*. Typhimurium (Figure 9), though the *in vivo* relevance of these findings awaits future animal studies.

**Figure 9:**
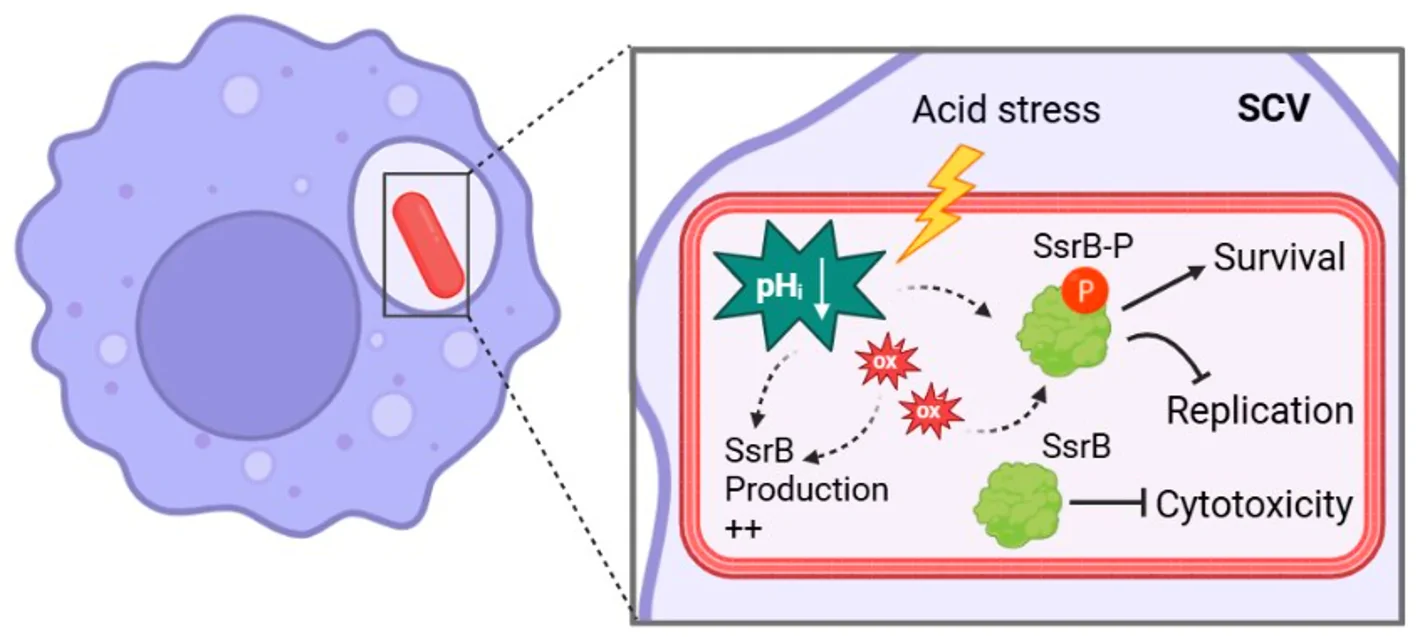
Multiple roles of SsrB during *S*. Typhi infection of THP-1 macrophages. Following infection of THP-1 cells, *S*. Typhi resides predominantly within the SCV, where its net intracellular replication is lower than that of *S*. Typhimurium, and escape into the host cytosol is limited. Within the SCV, the *S*. Typhi cytoplasm acidifies in response to the acidic vacuolar environment. Acidification is accompanied by a marked increase in the number of SsrB molecules per bacterium and by activation of the regulator, which drives SPI-2 gene expression. The arrows in this scheme summarize the genetic requirements established here and do not imply that the intervening steps have been identified. SsrB is required to limit cytotoxicity toward the host cell, a function that requires neither phosphorylation at D56 nor the H12-dependent increase in DNA-binding affinity. The targets through which SsrB acts were not determined in this study, and the blunt-ended arrow therefore denotes a phenotypic relationship rather than a defined repression event. Restriction of net intracellular replication has different requirements, since it depends on both phosphorylation at D56 and H12-dependent acid sensing, and is mediated in part by the SsrB-activated effector gene *sifB*. Whether the two outputs are executed by distinct or by shared downstream targets remains to be determined, and they are drawn as separate arrows only because their upstream requirements differ. *In vivo, S*. Typhi produces more SsrB than *S*. Typhimurium, while its more oxidizing intracellular environment reduces SsrB activity (Fernandez et al., 2025) and may also affect SsrB production. Together these serovar differences are likely to result in differential regulation of SsrB-dependent genes that influence net intracellular replication.

Using microscopy approaches, we provided evidence that *S*. Typhi intracellular burden was substantially reduced in macrophages compared to *S*. Typhimurium (Figure 2C). This observation correlated with flow cytometry experiments performed by Reuter et al. on *S*. Typhi strain 120130191 in comparison with *S*. Typhimurium NCTC12023 in the U937 macrophage model. In parallel, we also discovered that the multi-drug resistant strain H58, which is prevalent worldwide and especially in Africa and Oceania, reduces its replication in macrophages compared to the commonly employed Ty2 strain, as observed by both microscopy (Figure 1A) and gentamicin protection assays (Hamblin et al., 2023). Our results highlight phenotypic differences among the *S*. Typhi population. The low net intracellular replication of *S*. Typhi observed in our THP-1 macrophage infection model may represent a feature that could contribute to immune evasion and systemic dissemination, although this remains to be tested *in vivo*.

Acidification of *S*. Typhimurium is an essential event that activates the genetic cascade promoting bacterial survival within the hostile environment of the SCV (Chakraborty et al., 2015, 2017). Using two different approaches, we demonstrated that *S*. Typhi also acidified within the host. Vacuolar residence was inferred rather than measured for the FLIM population (Figure 3B), and the pHi values reported here should be understood as those of the acidified, vacuolar subpopulation that retains a fluorescent probe. While the pHi of an *ompR* null mutant was higher under neutral conditions, OmpR was not essential for *S*. Typhi to acidify its cytoplasm. Mechanisms of pH_i_ regulation therefore differ between the two serovars at both neutral and acidic pH. One practical consequence is that an *ompR* null strain cannot be used in *S*. Typhi as a genetically non-acidifying background, as it can in *S*. Typhimurium; separating the contribution of cytoplasmic pH from that of the regulators acting upon it requires a manipulation of pHi that is independent of both OmpR and the lysine decarboxylation system. This difference likely influences their mechanisms of pathogenesis.

Lower SPI-2 effector secretion has been reported in *S*. Typhi compared with *S*. Typhimurium (Fernandez et al., 2025; Reuter et al., 2021), and we demonstrate here that activation of the SsrB-dependent *sifA* promoter was correspondingly lower (Figure 4). This reduced expression was not due to a lack of acidification in *S*. Typhi (Figure 3). Using PALM, we tracked the number of SsrB molecules per bacteria in both Typhi and Typhimurium following macrophage infection. To our surprise, the number of SsrB molecules per bacteria was higher (>43% on average) in *S*. Typhi than in Typhimurium (Figure 5). In the case of SPI-2, this result indicates that the differential activation between *S*. Typhi and Typhimurium was not caused by a direct SsrB production defect in the Typhoidal strain, but rather depended on SsrB activity. While qRT-PCR would provide additional transcript-level data, this study relies on two orthogonal single-cell methods, the P_*sifA*_ reporter assay and PALM imaging, which measure SsrB activity at one promoter and SsrB abundance per bacterium, respectively. Because substantial cellular heterogeneity was observed, bulk mRNA measurements would likely obscure biologically relevant subpopulation differences. We previously demonstrated that the redox state upon macrophage infection was higher in *S*. Typhi than in *S*. Typhimurium, resulting in differences in *sifA* activation by SsrB (Fernandez et al., 2025). We hypothesized that other SsrB regulated genes were likely impacted by this redox change. Interestingly, oxidative stress also triggered the transcriptional activation of the *ssrA/B* operon in *S*. Typhimurium, by an action of PhoP and OmpR (Kim et al., 2022). We suspect that the redox differences between the two serovars induces a higher production of SsrB in *S*. Typhi.

While no phenotype was previously accounted for in the *S*. Typhi null strain in macrophages (Forest et al., 2010; Reuter et al., 2021), we report herein that SsrB is required for two distinct infection phenotypes during *S*. Typhi infection. Firstly, deletion of *ssrB* increased LDH release from infected macrophages, whereas the H12Q and D56A alleles did not. Control of cytotoxicity therefore requires the SsrB protein but requires neither phosphorylation at D56 nor the acid-dependent increase in DNA-binding affinity that depends on H12. One explanation for this pattern is that unphosphorylated SsrB acts as a repressor, as it does at *hilA* in biofilms, where the kinase SsrA is not active (Desai et al., 2019), and as has been proposed for SPI-1 in *S*. Typhimurium (Pérez-Morales et al., 2017). We emphasize that SPI-1 expression in *S*. Typhi was not measured in any of these backgrounds, and that the earlier study relied on overproduction of SsrB D56E, a variant that lacks DNA-binding activity (Liew et al., 2019). The behavior of the D56A allele toward SPI-1 has not been reported. Other explanations are equally consistent with our data. The *ssrB* null strain reaches a higher intracellular burden than the WT (Figure 2C and Supplementary Figure S3), and this alone may increase host cell damage; SsrB may also repress loci unrelated to SPI-1. The mechanism by which SsrB limits *S*. Typhi cytotoxicity therefore remains an open question. It can be resolved by monitoring SPI-1 promoter activity (for example P_*sipA*_, P_*sopB*_ or P_*hilA*_) in the WT, *ssrB* null, D56A and H12Q backgrounds during macrophage infection, and by testing whether removal of the SPI-1 injectisome suppresses the elevated LDH release of the *ssrB* null strain (van der Velden et al., 2000). We note that loss of *ssrB* reduced intracellular recovery only when *prgHIJ* was also deleted (Figure 2B), which indicates a genetic interaction between *ssrB* and SPI-1 in *S*. Typhi. This experiment does not distinguish SPI-1 derepression from the use of the SPI-1 injectisome for effector delivery (Hamblin et al., 2023). We did not determine the steady-state levels of the SsrB D56A and H12Q proteins in *S*. Typhi H58, and we cannot formally exclude a partial reduction in abundance. Several observations nonetheless argue against a destabilized protein as the explanation for the phenotypes reported here. Both alleles are single-codon substitutions at the native chromosomal locus, expressed from the native promoter, so the two strains differ only by the substituted codon. Both retain wild-type-level control of host cytotoxicity, a function that is lost in the *ssrB* null strain, thus neither behaves as a functional null. The H12Q protein has been characterized with purified material and retains wild-type-like DNA-binding affinity and near-full transcriptional activity at neutral pH while remaining phosphorylation-competent, and His12 was identified as the pH-sensing residue by a systematic screen of receiver-domain histidines (Shetty and Kenney, 2023). Direct measurement would require PAmCherry-tagged versions of both alleles, counted by PALM as we did here with wild-type SsrB. Interestingly, both SsrB~P and acid-dependent DNA binding act to reduce *S*. Typhi net intracellular replication in the THP-1 model, since D56A and H12Q were both required (Figure 7C). One of the genes involved is *sifB*, but it is obvious that other genes are required for this phenotype. It is possible that SifB has different effects depending on the host cell type, or it could be a serovar-specific effect. SifB was co-localized to the SCV with SifA and SseJ, suggesting a probable interaction between these effectors. It is known that SPI-2 effectors such as *sifA* or *sseJ*, respectively, promote or reduce *S*. Typhimurium growth by modulating endosomal trafficking for the bacteria to acquire nutrients. *sseJ* is a pseudogene in *S*. Typhi (Trombert et al., 2010), thus its absence may impact SifB activity or localization.

The SsrB regulon in *S*. Typhimurium involves numerous genes implicated in metabolism and growth, such as *guaAB*, that SsrB~P represses by binding to the *guaA* ATG codon. Interestingly, those genes are differentially regulated *in vivo* between *S*. Typhi (Faucher et al., 2006) and *S*. Typhimurium (Hautefort et al., 2008), i.e. the *guaAB* transcription is decreased (*S*. Typhi) or increased (*S*. Typhimurium) upon entry into macrophages. We hypothesize that differences we have uncovered between the two serovars, including: the number of SsrB molecules and the differences in the redox environment might contribute to these differences in gene expression, although this hypothesis requires further investigation (Fernandez et al., 2025) and experiments herein). In the case of *sifA*, oxidation of SsrB prevents or reduces promoter binding. Other SsrB-dependent promoters may be differentially sensitive, since they differ in SsrB-binding affinity, in the number of SsrB boxes and in the extent of H-NS silencing. Reporters that could test this include: P_*ssaG*_, P_*ssaB*_ and P_*steC*_. P_*steC*_ is of particular interest because *steC* was not activated in *S*. Typhi within macrophages (Faucher et al., 2006).

We cannot rule out that deletion of *ssrB* led to the up-regulation of SPI-1 (Pérez-Morales et al., 2017). However, it is clear from the results presented herein and from (Hamblin et al., 2023) that several SPI-2 effectors (such as SifA), whose activation depends on SsrB, are required for *S*. Typhi survival in THP-1 cells. Taken together, we demonstrate that the gentamicin protection assay has important limitations for assessing SsrB function in *S*. Typhi, as it does not account for differential host cell cytotoxicity. The apparent lack of phenotype in earlier gentamicin protection studies likely reflects suboptimal experimental parameters (e.g., MOI, infection duration, or readout timing) rather than a true absence of SsrB function, and appears to contradict the drastic activation of SsrB we observed by microscopy (Figure 5).

Our data establish that the two phenotypes have different genetic requirements at the level of SsrB. Limiting host cytotoxicity requires only the SsrB protein, whereas restricting net intracellular replication additionally requires phosphorylation at D56 and the H12-dependent increase in DNA-binding affinity at acid pH. The D56A and H12Q alleles therefore separate the two readouts, since they reproduce the replication phenotype of the *ssrB* null strain without reproducing its cytotoxicity phenotype. What was not established is whether the two phenotypes are executed by distinct downstream targets. SsrB controls a large regulon, and it remains possible that a shared set of targets influences both readouts, or that the higher bacterial burden of the *ssrB* null strain contributes directly to the additional host cell damage. Ideally, identifying a target whose loss reproduces one phenotype but not the other would clarify the SsrB behavior we observed. As a result, we describe the effects of SsrB as pleiotropic, with two genetically separable input requirements, rather than as two mechanistically independent activities.

## Data Availability

The raw data supporting the conclusions of this article will be made available by the authors upon reasonable request.

## Supplementary material

Supplementary Figure S1 *S*. Typhi WT and an *ompR* null strain acidify their cytosol in response to an acidic environment Intracellular pH of *S*. Typhi was investigated using BCECF and mCherryTYG FLIM. **(A)** Bacteria (Blue and red lines) were clamped at different pH values to construct a standard curve. The standard curve overlaps with BCECF dye (black dots) incubated in buffers at various pH values. Data are presented as mean ± SEM (*n* = 50 bacteria per pH condition). **(B)** Intracellular pH of *S*Ty H58 strains was measured *in vitro* (MgM pH 7.2 or 5.6), as well in infected THP-1 cells. Lifetime values of individual bacteria, represented by dots, were converted into pHi using the standard curve in **(A)**. Both WT and *ompR* null strains respond to an acidic shift by acidifying the bacterial cytosol. The pHi for the *ompR* null strain was higher on average than the WT, even under neutral conditions. *n* = 25 bacteria per pH. ^***^*p* < 0.001, Student's *t*-Test. The median values are indicated in red with the corresponding value annotated. **(C)** The pHi of the H58 *ompR* null strain was investigated using mCherryTYG and FLIM (see Section 2), during infection of THP-1 cells. Lifetime values from individual bacteria were converted to intracellular pH_i_. The H58 *ompR* null strain acidifies *in vivo* over time. *n* = 8–25 bacteria per pH.

Supplementary Figure S2 Analysis of SsrB levels in *S*. Typhi in response to acid pH *in vitro* Molecular counting of SsrB molecules in *S*Ty H58 (Δ*ssrB*::*kan attB*::PAmCherry-ssrB) in response to acidic pH *in vitro*. Bacteria were grown in neutral or acid LB broth until exponential phase (OD_600_~0.6) and stationary phase (OD_600_~1.5), bacteria were then fixed and imaged using PALM. The average number of SsrB molecules per cell at pH 4.5 exponential phase was 41, at pH 7.4 exponential phase, it was 32; in pH 4.5 stationary phase the average was 55, and at pH 7.4 stationary phase, it was 30. Each dot represents a single bacterium. For exponential-phase bacteria, *n* = 107 and 192 bacteria for pH 4.5 and pH 7.2, respectively; for stationary-phase bacteria, *n* = 182 and 122 bacteria for pH 4.5 and pH 7.2, respectively. ^****^*p* < 0.0001, Student's *t*-test.

Supplementary Figure S3 The acid response driven by SsrB reduces net intracellular replication in THP-1 cells The number of *S*Ty H58 pFPv-mCherry per infected THP-1 cell was enumerated by confocal microscopy at 24 hpi. The strains used were *S*. Typhi STy H58 (WT), and its *ssrB* null strain or strains expressing mutant SsrB (H12Q or D56A). Each dot represents a single infected THP-1 cell (*n* = 100 infected cells). ^*^*p* < 0.05, ^****^*p* < 0.0001 vs. WT strain, One-way ANOVA.

Supplementary Table S1 Oligonucleotide primers used in this study.

Supplementary Table S2 Effect sizes for the quantitative phenotypes reported in this study.
