# Supplementary material for: The multifaceted role of SsrB in S. Typhi H58 during THP-1 macrophage infection
Source: Front Microbiol. 2026 Oct 12;17:1849419. doi: 10.3389/fmicb.2026.1849419 (PMC13696444; doi:10.3389/fmicb.2026.1849419)
Supplement: Supplementary file 1 [file Data_Sheet_1.zip › Supplementary matterial presentation/Table S2.docx]

**Table S2. Effect sizes for the quantitative phenotypes reported in this study**

Effect sizes are reported as fold change relative to the wild-type or control condition, as a percentage difference, or, for pH and promoter-activity measurements, as an absolute difference on the measured scale. Significance symbols are those shown in the corresponding figure panel: *p < 0.05, **p < 0.01, ***p < 0.001, ****p < 0.0001; ns, not significant.

| **Phenotype (panel)** | **Comparison** | **Effect size** | **n** | **Test / reported significance** |
| --- | --- | --- | --- | --- |
| **Uptake (0.5 hpi)** | | | | |
| Bacterial uptake (Fig. 6B) | ΔssrB vs WT | 1.16 × WT | 4 indep. infections × 4 tech. repl. | One-way ANOVA, ns |
| Bacterial uptake (Fig. 6B) | ssrB H12Q vs WT | 0.98 × WT | 4 indep. infections × 4 tech. repl. | One-way ANOVA, ns |
| Bacterial uptake (Fig. 6B) | ssrB D56A vs WT | 0.94 × WT | 4 indep. infections × 4 tech. repl. | One-way ANOVA, ns |
| Bacterial uptake (Fig. 6B) | ΔsifB vs WT | 1.06 × WT | 4 indep. infections × 4 tech. repl. | One-way ANOVA, ns |
| **Intracellular burden (bacteria per infected cell, single-cell microscopy)** | | | | |
| Burden 24 hpi (Fig. 1A) | STy Ty2 vs STy H58 | mean 5 vs 2 bacteria/cell | 100 cells/condition, 3 exp. | One-way ANOVA, **** |
| Burden 24 hpi (Fig. 1A) | STm 14028s vs STy H58 | mean 8 vs 2 bacteria/cell; maximum >50 vs ~20 | 100 cells/condition, 3 exp. | One-way ANOVA, **** |
| Burden 24 hpi (Fig. 2C) | H58 ΔssrB vs H58 WT | small but significant increase in median; maximum 18 (WT) vs 26 (ΔssrB) bacteria/cell | 100 cells/condition, 3 exp. | One-way ANOVA, * |
| Burden 24 hpi (Fig. 2C) | 14028s ΔssrB vs 14028s WT | maximum 22 vs 76 bacteria/cell | 100 cells/condition, 3 exp. | One-way ANOVA, *** |
| Burden 24 hpi (Fig. S3) | ΔssrB vs WT | median unchanged (~2 bacteria/cell); upper tail extended, maximum 27 vs 18 | 100 infected cells/condition | One-way ANOVA, * |
| Burden 24 hpi (Fig. S3) | ssrB H12Q vs WT | median unchanged (~2 bacteria/cell); upper tail extended, maximum 41 vs 18 | 100 infected cells/condition | One-way ANOVA, * |
| Burden 24 hpi (Fig. S3) | ssrB D56A vs WT | median ~4 vs ~2 bacteria/cell; maximum 26 vs 18 | 100 infected cells/condition | One-way ANOVA, **** |
| Burden 24 hpi (Fig. 8B) | ΔsifB vs WT | median ~3 vs ~2 bacteria/cell; maximum 45 vs 27 | 100 infected cells/condition, 3 exp. | Student's t-test, * |
| **Net intracellular replication (CFU 24 hpi / 0.5 hpi, normalised to WT)** | | | | |
| Net replication (Fig. 2B) | ΔssrB vs WT | 1.02 × WT (+2%) | 5 indep. exp. × 4 tech. repl. | One-way ANOVA, ns |
| Net replication (Fig. 2B) | ΔprgHIJ vs WT | 0.72 × WT (−28%); spread wide, interval includes 1.0 | 5 indep. exp. × 4 tech. repl. | One-way ANOVA, ns |
| Net replication (Fig. 2B) | ΔprgHIJ ΔssrB vs WT | 0.39 × WT (−61%) | 5 indep. exp. × 4 tech. repl. | One-way ANOVA, **** |
| Net replication (Fig. 7C) | ΔssrB vs WT | 1.02 × WT (+2%) | 3 indep. infections × 4 tech. repl. | One-way ANOVA, ns |
| Net replication (Fig. 7C) | ssrB H12Q vs WT | 1.37 × WT (+37%) | 3 indep. infections × 4 tech. repl. | One-way ANOVA, *** |
| Net replication (Fig. 7C) | ssrB D56A vs WT | 1.32 × WT (+32%) | 3 indep. infections × 4 tech. repl. | One-way ANOVA, * |
| Net replication (Fig. 8A) | ΔsifB vs WT | 1.08 × WT (+8%) | 5 indep. exp. × 4 tech. repl. | Student's t-test, * |
| Net replication (Fig. 8D) | ssrB H12Q + constitutive sifB vs ssrB H12Q | 1.38 → 1.13 × WT | 3 indep. infections × 4 tech. repl. | One-way ANOVA, ns (complemented strain also ns vs WT) |
| Net replication (Fig. 8D) | ssrB D56A + constitutive sifB vs ssrB D56A | 1.31 → 0.83 × WT | 3 indep. infections × 4 tech. repl. | One-way ANOVA, * |
| **Competitive index in co-infection (mutant / WT, 24 hpi)** | | | | |
| Competitive index (Fig. 7D) | ΔssrB vs WT | 1.27 | 3 indep. infections, 50 co-infected cells each | One-way ANOVA, * |
| Competitive index (Fig. 7D) | ssrB H12Q vs WT | 1.30 | 3 indep. infections, 50 co-infected cells each | One-way ANOVA, * |
| Competitive index (Fig. 7D) | ssrB D56A vs WT | 1.32 | 3 indep. infections, 50 co-infected cells each | One-way ANOVA, ** |
| Competitive index (Fig. 8C) | ΔsifB vs WT | 1.38 | 3 indep. infections, 50 cells each | Student's t-test, * |
| **Host cell cytotoxicity (LDH release, 24 hpi)** | | | | |
| Cytotoxicity, absolute (Fig. 6A) | ΔssrB vs WT | 26% vs 17% LDH release (ratio 1.5) | 3 indep. infections × 4 tech. repl. | Two-way RM ANOVA, p = 0.087 |
| Cytotoxicity, normalised (Fig. 7A) | ΔssrB vs WT | 1.65 × WT (+65%) | 5 indep. exp. × 4 tech. repl. | One-way ANOVA, **** |
| Cytotoxicity, normalised (Fig. 7A) | ssrB H12Q vs WT | 0.87 × WT | 5 indep. exp. × 4 tech. repl. | One-way ANOVA, ns |
| Cytotoxicity, normalised (Fig. 7A) | ssrB D56A vs WT | 1.00 × WT | 5 indep. exp. × 4 tech. repl. | One-way ANOVA, ns |
| **Bacterial cytoplasmic pH (mCherryTYG FLIM; effect size as ΔpH units)** | | | | |
| pHi in vitro (Fig. 3A) | MgM pH 5.6 vs 7.2 | 6.63 vs 6.98 (ΔpH −0.35) | 50–60 bacteria, 3 exp. | Student's t-test, **** |
| pHi in vitro (Fig. 3A) | LB pH 4.5 vs 7.0 | 6.10 vs 6.73 (ΔpH −0.63) | 50–60 bacteria, 3 exp. | Student's t-test, **** |
| pHi in THP-1 (Fig. 3A) | 24 hpi vs inoculum | 5.68 vs 6.82 (ΔpH −1.14) | 50–60 bacteria, 3 exp. | One-way ANOVA, *** |
| pHi in vitro, BCECF (Fig. S1B) | ΔompR vs WT at pH 7.2 | 7.30 vs 6.81 (ΔpH +0.49) | 25 bacteria per condition | Student's t-test, *** |
| **SsrB abundance (PALM molecular counting, molecules/µm²)** | | | | |
| SsrB abundance (Fig. 5B) | STy H58, 24 hpi vs inoculum | ~10-fold increase | 45–50 bacteria, 3 exp. | One-way ANOVA, **** |
| SsrB abundance (Fig. 5B) | STy H58 vs STm 14028s, 24 hpi | 160 vs 125 molecules/µm² (means); reported in the text as >43% higher on average — see note 5 | 45–50 bacteria, 3 exp. | One-way ANOVA, ** |
| SsrB abundance in vitro (Fig. S2) | LB pH 4.5 vs 7.4, exponential | 41 vs 32 molecules (1.28-fold) | 107 and 192 bacteria | Student's t-test, **** |
| SsrB abundance in vitro (Fig. S2) | LB pH 4.5 vs 7.4, stationary | 55 vs 30 molecules (1.83-fold) | 182 and 122 bacteria | Student's t-test, **** |
| **SsrB-dependent PsifA promoter activity (BFP/mCherry ratio)** | | | | |
| PsifA in vitro (Fig. 4A) | WT, pH 4.5 vs 7.0 | 0.82 vs 0.35 (2.3-fold) | ~50 bacteria per condition | One-way ANOVA, **** |
| PsifA in vitro (Fig. 4A) | ΔssrB, pH 4.5 vs 7.0 | 0.48 vs 0.40 (1.2-fold) | ~50 bacteria per condition | One-way ANOVA, ns |
| PsifA in vitro (Fig. 4A) | WT + cadBA, pH 4.5 vs 7.0 | 0.18 vs 0.34 (0.5-fold) | ~50 bacteria per condition | One-way ANOVA, *** |
| PsifA in THP-1 (Fig. 4B) | STm 14028s vs STy H58, 24 hpi | median 2.17 vs 1.29 (1.7-fold); >90% vs ~70% of bacteria activating | 50 bacteria pooled across 3 indep. infections | Student's t-test, **** |
