# Supplementary material for: The multifaceted role of SsrB in S. Typhi H58 during THP-1 macrophage infection
Source: Front Microbiol. 2026 Oct 12;17:1849419. doi: 10.3389/fmicb.2026.1849419 (PMC13696444; doi:10.3389/fmicb.2026.1849419)
Supplement: Supplementary file 1 [file Data_Sheet_1.zip › Supplementary matterial presentation/Table S1.docx]

**Table S1. Oligonucleotide primers used in this study.**

| **Primer name** | **Sequence (5′→3′)** | **Purpose** |
| --- | --- | --- |
| ssrB_ko_FP (forward) | AGAATATAAGATCTTATTAGTAGACGATCATGAAATCATCGTGTAGGCTGGAGCTGCTTC | *ssrB* knockout in H58 WT by λ-Red recombination |
| ssrB_ko_RP (reverse) | GTAACTCTGTCACTTTATGAACCTGTAGCTTTCTCATCATCATATGAATATCCTCCTTAG | *ssrB* knockout in H58 WT by λ-Red recombination |
| ssrB_chk_FP (forward) | ACTTATCCTTGATCTTAGCT | Confirmation of *ssrB* knockout |
| ssrB_chk_RP (reverse) | TATGGATTTTGTCGATGATG | Confirmation of *ssrB* knockout |
| ssrB H12Q  (forward) | TTAGTAGACGATCATGAAATCATCATTAA | Mutation of the lab stock pKD3-ssrB. Used with reverse complemented |
| ssrB D56A  (forward) | CAGGTAGACTAAGAGCAAGGATAAGTATGTC | Mutation of the lab stock pKD3-ssrB. Used with reverse complemented |
| ssrB mut HR  (Forward) | ATTACTTAATATTATCTTAATTTTCGCGAGGGCAGCAAAATGAAAGAATA | Amplification of the ssrB H12Q or D56A mutant gene fused to the ATB cassette (from pKD3-ssrB lab stock Shetty 2023 ) for homologous recombination ate the locus |
| ssrB mut HR  (reverse) | CAAAATATGACCAATGCTTAATACCATCGGACGCCCCTGGGTGTAGGCTGGAG | Amplification of the ssrB H12Q or D56A mutant gene fused to the ATB cassette (from pKD3-ssrB lab stock Shetty 2023 ) for homologous recombination ate the locus |
| sifB_ko_FP (forward) | AAGATATTCCGTTATGTATTATGATACGTCAGGATCACTAGTGTAGGCTGGAGCTGCTTC | *sifB* knockout in H58 WT by λ-Red recombination |
| sifB_ko_RP (reverse) | AGGCTGTTATGAATTTGTAGCGCTACATATTCAGTAAGCTCATATGAATATCCTCCTTAG | *sifB* knockout in H58 WT by λ-Red recombination |
| sifB_chk_FP (forward) | TATCATCACCATCATGCAGA | Confirmation of *sifB* knockout |
| sifB_chk_FP (revers) | CATAGTAAATCCATTATTCC | Confirmation of *sifB* knockout |
| prjHIJ ko_RP (forward) | CTGTGCGGTAATCTGCTGCTATCGAGAACGACAGATATCGTTAAGACCCACTTTCACATT | *prjHIJ* knockout in H58 WT by λ-Red recombination |
| prjHIJ _ko_RP (reverse) | CATCTCTTGCGAAATAGCCAGCTCATTAGGATCCGTCACCCTAAGCACTTGTCTCCTG | *prjHIJ* knockout in H58 WT by λ-Red recombination |
| *prjHIJ* _chk_FP (forward) | AGGACGCTATGTGCAGTTTG | Confirmation of *prjHIJ* knockout |
| prjHIJ chk_RP (reverse) | CAGGGACAATAGTTGCAATC | Confirmation of *prjHIJ* knockout |
| cadBA PCR  (Forward) | TTTAAGAAGGAGATGGTACCATGAGTTCTGTCAAAAAGAT | Amplification of *cadBA* from gDNA  Construction of constitutive pTet-cadBA |
| cadBA PCR  (Reverse) | ATTTGATGCCTCTAGACTCACATAAAGTCTCCTTGCTGCG | Amplification of *cadBA* from gDNA  Construction of constitutive pTet-cadBA |
| pZS PCR  (Forzward) | CGCAGCAAGGAGACTTTATGTGAGTCTAGAGGCATCAAAT | Amplification of *plasmid* pZS backbone without ATB  Construction of constitutive pTet-cadBA |
| pZS PCR  (Reverse) | GATCTTGAAGTTCCTATTCCTAACTGTCAGACCAAGTTTA | Amplification of *plasmid* pZS backbone without ATB  Construction of constitutive pTet-cadBA |
| Kn PCR  (Forward) | TAAACTTGGTCTGACAGTTAGGAATAGGAACTTCAAGATC | Amplification of *plasmid* Kn cassette from pUC18R6KT  Construction of constitutive pTet-cadBA |
| Kn PCR  (reverse) | TGTTTATTTTTCTAAATACAAGAGCGCTTTTGAAGCTGAT | Amplification of *plasmid* Kn cassette from pUC18R6KT  Construction of constitutive pTet-cadBA |
| OmpR mutant and pZS-sifA are from Fernandez et.al, 2025 | | |
